# Collaborative Duality of CircGLIS3(2) RNA and Protein in human Wound Repair

**DOI:** 10.1002/advs.202416784

**Published:** 2025-04-25

**Authors:** Guanglin Niu, Maria A. Toma, Jennifer Geara, Xiaowei Bian, Yongjian Chen, Lihua Luo, Qizhang Wang, Yunting Xiao, Manika Vij, Minna Piipponen, Zhuang Liu, Sho Oasa, Letian Zhang, Dörte Schlesinger, Ákos Végvári, Dongqing Li, Aoxue Wang, Vladana Vukojević, Simon J Elsässer, Pehr Sommar, Ning Xu Landén

**Affiliations:** ^1^ Dermatology and Venereology Division Department of Medicine Solna Center for Molecular Medicine Karolinska Institutet Stockholm 17176 Sweden; ^2^ Department of Oromaxillofacial Head and Neck Oncology Shanghai Ninth People's Hospital College of Stomatology Shanghai Jiao Tong University School of Medicine Shanghai 200025 China; ^3^ Key Laboratory of Basic and Translational Research on Immune‐Mediated Skin Diseases Chinese Academy of Medical Sciences Jiangsu Key Laboratory of Molecular Biology for Skin Diseases and STIs Institute of Dermatology Chinese Academy of Medical Sciences and Peking Union Medical College Nanjing 210003 China; ^4^ Department of Clinical Neuroscience Center for Molecular Medicine Karolinska Institutet Stockholm 17176 Sweden; ^5^ Science for Life Laboratory Department of Medical Biochemistry and Biophysics Division of Genome Biology Karolinska Institutet Stockholm 17165 Sweden; ^6^ Division of Chemistry I Department of Medical Biochemistry and Biophysics Karolinska Institutet Stockholm 17177 Sweden; ^7^ Department of Dermatology The Second Hospital of Dalian Medical University College of Integrative Medicine Dalian Medical University Dalian 116021 China; ^8^ Department of Plastic and Reconstructive Surgery Karolinska University Hospital Stockholm 17176 Sweden; ^9^ Present address: State Key Laboratory of Oral Diseases National Clinical Research Center for Oral Diseases Department of Oral and Maxillofacial Surgery West China Hospital of Stomatology Sichuan University Chengdu 610041 China; ^10^ Present address: Department of Dermatology and Venereology Medical Center – University of Freiburg 79110 Freiburg Germany

**Keywords:** CircRNA, coding, non‐coding, skin wound healing

## Abstract

The discovery of an increasing number of translatable circular RNAs (circRNAs) raises the question of whether their coding and non‐coding functions can coexist within the same cell. This study profiles the dynamic expression of circRNAs during human skin wound healing. *CircGLIS3(2)* is identified, a circRNA whose levels transiently rise in dermal fibroblasts of acute wounds and are abnormally overexpressed in keloids, a fibrotic skin condition. Injury signals such as IL‐1α, TGF‐β, hypoxia, and ER stress induce both expression and cap‐independent translation of CircGLIS3(2). The RNA form of *CircGLIS3(2)* activates fibroblasts into matrix‐secreting cells, while its encoded protein promotes cell proliferation, collectively enhancing wound repair. Mechanistically, *CircGLIS3(2)* RNA stabilizes the cytoplasmic protein PCOLCE, while its protein binds to BTF3 in the nucleus. Both the RNA and protein are essential for wound closure in human and murine models. *CircGLIS3(2)*’s bifunctional nature expands its functional spectrum, improving cellular adaptability during environmental changes and offering a promising therapeutic target for wound repair and scar reduction.

## Graphical abstract

CircGLIS3(2), a circRNA transiently upregulated in dermal fibroblasts during acute wound healing, is induced by IL‐1α and TGF‐β. Its RNA activates fibroblasts into matrix‐secreting cells, while its encoded protein enhances proliferation. The RNA stabilizes PCOLCE, and the protein interacts with BTF3, collectively accelerating wound repair.

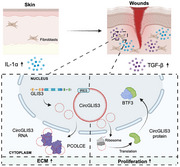

## 1 Introduction

Impaired tissue repair can result in chronic wounds and excessive scarring, imposing a substantial medical and economic burden worldwide.^[^ ^1^ ^]^ To effectively address these complications, understanding the molecular mechanisms of healthy skin repair is essential. Wound healing progresses through three overlapping phases—inflammation, growth, and remodeling— orchestrated by complex signaling pathways and coordinated interactions among various cell types.^[^ ^2^ ^]^ Dermal fibroblasts are key players in skin repair; they proliferate, migrate, synthesize extracellular matrix (ECM), promote wound contraction, and secrete growth factors and cytokines that facilitate communication with other cell types.^[^ ^3^ ^]^ A comprehensive grasp of fibroblast functional regulation during healing can offer valuable insights into wound pathologies and informs novel therapeutic strategies.

Circular RNAs (circRNAs) are covalently closed RNA molecules formed by back‐splicing.^[^ ^4^ ^]^ While many circRNAs have been found to play significant physiological and pathological roles, their involvement in skin wound healing remains underexplored.^[^ ^3^ ^]^ Traditionally considered non‐coding, circRNAs are known to function through mechanisms such as miRNA sponging, protein interactions, and transcriptional regulation.^[^ ^5^ ^]^ Recently, some endogenous circRNAs have been shown to encode novel proteins or isoforms through cap‐independent translation, driven by internal ribosome entry sites (IRES) or N6‐methyladenosine (m6A) sequences.^[^ ^6^ ^]^ However, whether these translatable circRNAs can simultaneously perform both RNA and protein functions within the same cell or tissue is still an open question.

To investigate circRNAs in wound repair, we profiled circRNA expression during human skin wound healing and discovered *CircGLIS3(2)* as transiently upregulated in dermal fibroblasts of acute wounds. Uniquely, *CircGLIS3(2)* exhibits dual functionality: it not only acts as an RNA but also encodes a 131‐amino‐acid protein. The RNA form activates fibroblasts into matrix‐secreting cells by stabilizing the PCOLCE protein in the cytoplasm, while the encoded protein promotes cell proliferation by interacting with BTF3 protein in the nucleus, collectively enhancing wound repair. *CircGLIS3(2)* thus expands its functional spectrum through both its RNA and protein forms, bolstering cellular adaptability during rapid environmental changes.

## 2 Results

### 2.1 *CircGLIS3(2)* Expression is Upregulated in Human Wound Fibroblasts

**Figure 1:**
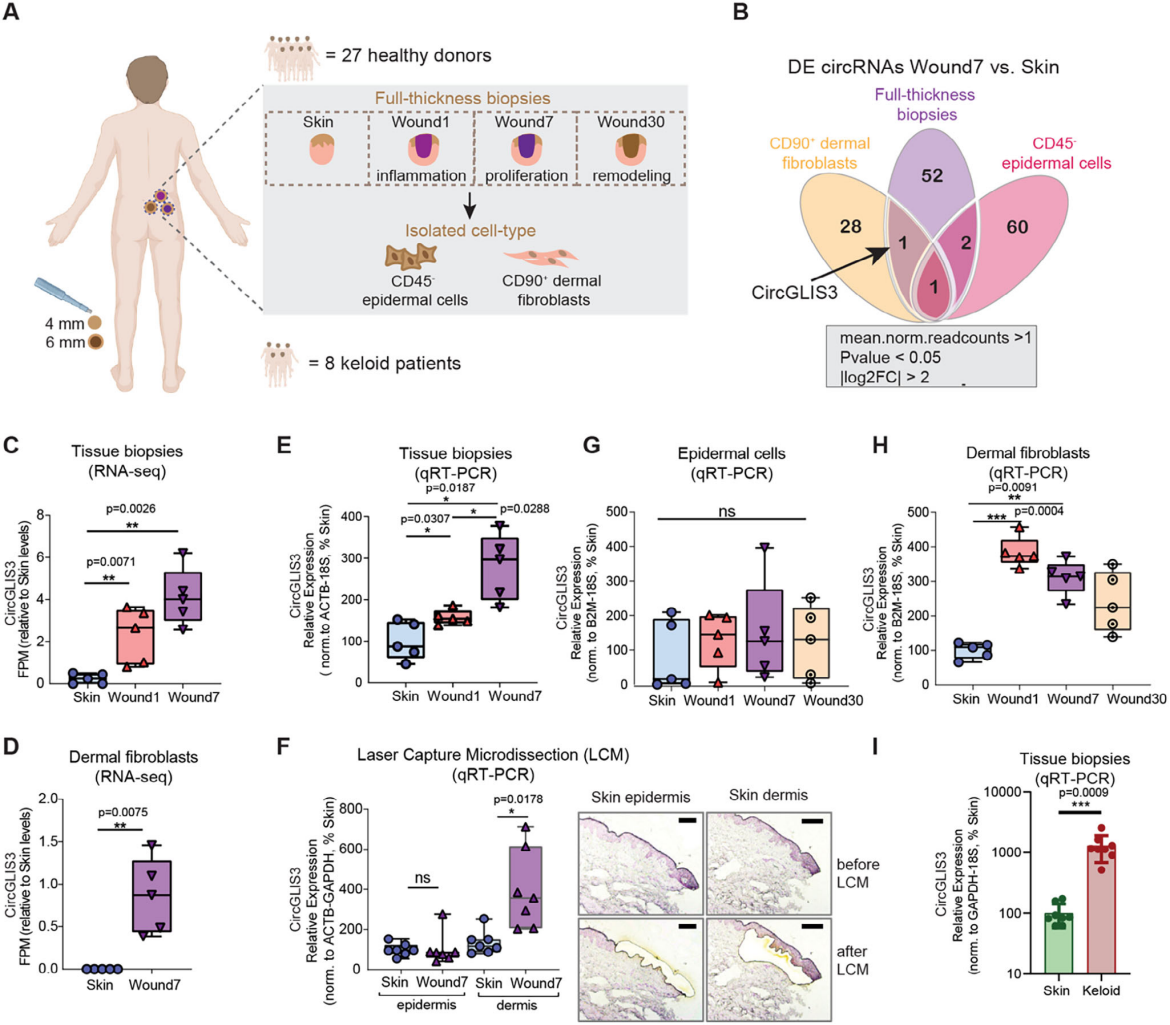
*CircGLIS3(2)* expression is upregulated in human wound fibroblasts. A) Excisional wounds were created on the skin of 27 healthy volunteers and collected 1 (Wound1), 7 (Wound7), and 30 days later (Wound30) from the same donor. CD45^−^ epidermal cells and CD90^+^ fibroblasts were isolated from matched skin and Wound7 samples. Biopsies were also collected from lesional sites and surrounding skin of 8 keloid patients. CircRNAs were analyzed in these clinical samples by RNA‐seq, qRT‐PCR, and laser capture microdissection (LCM). B) Venn diagram showing the commonly identified differentially expressed (DE) circRNAs in the isolated cell types and tissue biopsies of the skin and Wound7 analyzed by RNA‐seq. *CircGLIS3(2)* expression in the skin and wound biopsies (n = 5 donors) C) and isolated fibroblasts (n = 5 donors) D) was analyzed by RNA‐seq. qRT‐PCR validation of *CircGLIS3(2)* expression in additional skin and wound biopsies (n = 5 donors) E), epidermal and dermal compartments of skin and wounds separated by LCM (n = 7 donors) F), CD45^−^ epidermal cells G) and CD90^+^ fibroblasts H) from skin and wounds (n = 5 donors), donor matched skin and keloid biopsies (n = 8 donors) I). Data are presented as means ± SD. ns P ≥ 0.05, *P< 0.05, **P< 0.01 and ***P< 0.001 by Wilcoxon test (C, D), or one‐way ANOVA and Tukey's multiple comparisons test (G, H), or Paired t test (E, F and I).

To investigate gene expression regulation in human tissue repair, we developed a unique human wound healing model. Full‐thickness excisional wounds were created in healthy volunteers, and wound tissue was collected at 1, 7, and 30 days post‐injury to capture the inflammation, proliferation, and remodeling phases (**Figure** **1**A and Tables S1 and S2, Supporting Information).^[^ ^7^ ^]^ CD45^−^ epidermal keratinocytes and CD90^+^ dermal fibroblasts were isolated from these biopsies using magnetic‐activated cell sorting (MACS). We performed total RNA sequencing (RNA‐seq) on donor‐matched skin and day 7 wound tissues (n = 5 donors), as well as isolated cells (n = 5 donors), revealing differentially expressed circRNAs in tissue biopsies, purified keratinocytes, and fibroblasts (mean normalized read counts > 1, |log2FoldChange| > 2, P < 0.05 by Wald test, Figure 1B and Table S3, Supporting Information). Notably, *CircGLIS3(2)* emerged as the circRNA with increased levels in wound fibroblasts and whole biopsies, while remaining unchanged in keratinocytes (Figure 1B–D and Table S3, Supporting Information).

**Figure 2:**
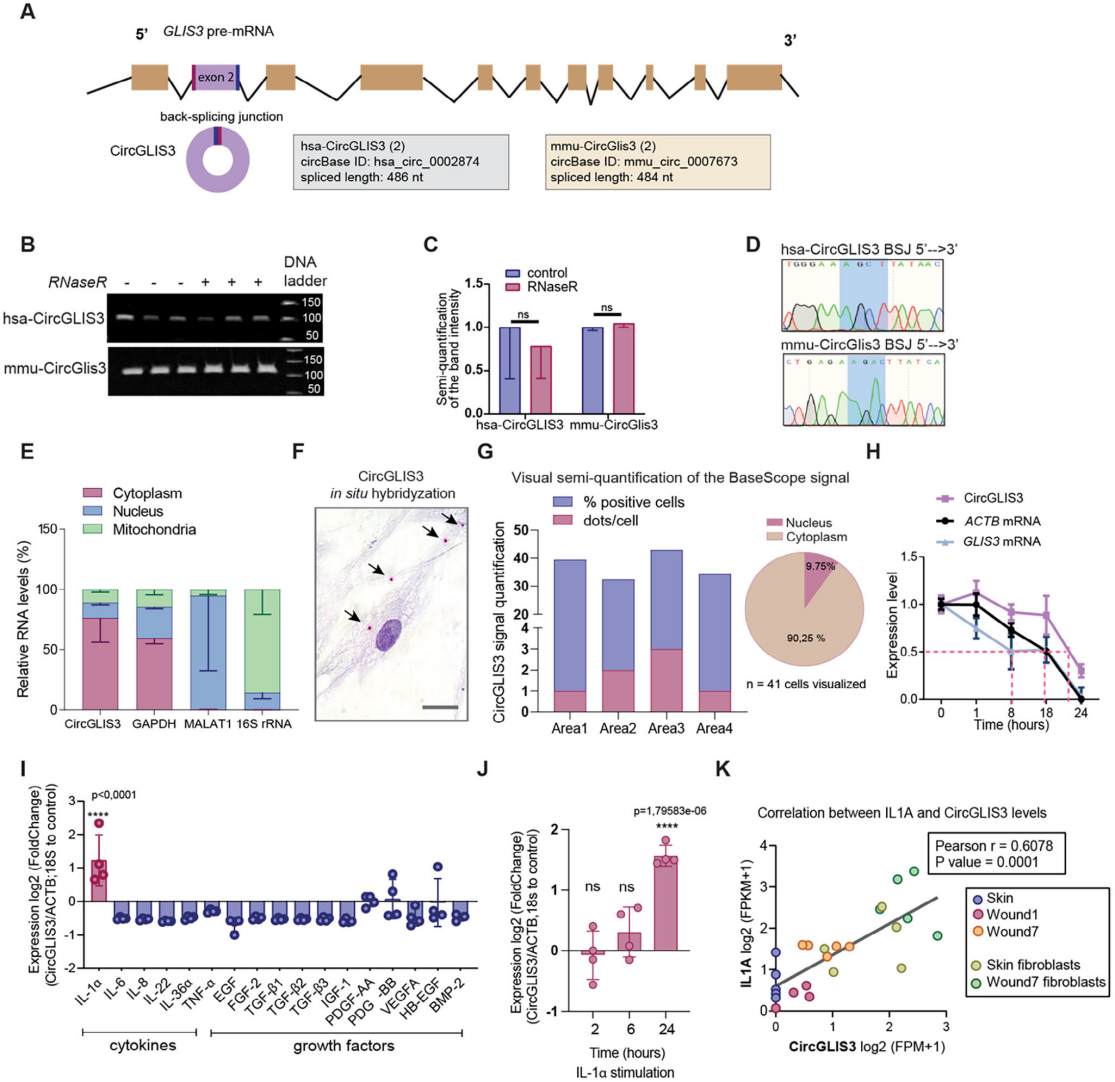
Molecular characterization of *CircGLIS3(2)*. A) Illustration of *CircGLIS3(2)* biogenesis. B) Agarose gel electrophoresis of *CircGLIS3(2)* RT‐PCR products from RNaseR‐digested or control RNA from human (top) and mice (bottom) fibroblasts (n = 3). Band intensity was quantified in C). D) Sanger sequencing of the RT‐PCR products. E) qRT‐PCR of *CircGLIS3(2)*, *GAPDH*, *MALAT1*, and *16S* rRNA in nuclear, cytoplasmic, and mitochondrial fractions of human fibroblasts (n = 3). F) In situ hybridization of *CircGLIS3(2)* in human fibroblasts. Scale bar = 20 µm G) Visual semi‐quantification of the *CircGLIS3(2)* positive cells and the number of *CircGLIS3(2)* signal dots/cell. H) qRT‐PCR of *CircGLIS3(2)*, *GLIS3* mRNA, and *ACTB* mRNA levels in human fibroblasts treated with Actinomycin‐D (n = 4). qRT‐PCR of *CircGLIS3(2)* in human fibroblasts treated with wound‐related cytokines and growth factors for 24 h (n = 4) I), or IL‐1α for 2–24 h (n = 4) J). K) Correlation between *CircGLIS3(2)* and *IL1A* expression in human wound samples analyzed by RNA‐seq. Data are presented as means ± SD. ns P ≥ 0.05 and ****P<0.0001 by Student's t‐test (C, J) or one‐way ANOVA and Tukey's multiple comparisons test (I) or Pearson's correlation test (K).

We further validated *CircGLIS3(2)* levels in additional clinical samples using quantitative real‐time PCR (qRT‐PCR) with divergent primers to specifically amplify the back‐splicing junction (BSJ) of *CircGLIS3(2)*, absent in its linear isoform (**Figure** **2**A). qRT‐PCR was performed on full‐thickness skin and wound tissues (Figure 1E), epidermal and dermal compartments separated by laser capture microdissection (LCM) (Figure 1F), and on isolated keratinocytes and fibroblasts from donor‐matched skin and wounds at each healing phase (Figure 1G,H). The results consistently confirmed significant upregulation of *CircGLIS3(2)* in dermal fibroblasts during wound healing. Notably, *CircGLIS3(2)* was also overexpressed in keloids, a fibroproliferative skin disease, compared to donor‐matched normal skin (Figure 1I and Tables S1 and S2, Supporting Information). Given its abundance in wound fibroblasts and keloids, we further investigated *CircGLIS3(2)*’s function in dermal fibroblasts.

### 2.2 Molecular Characterization of *CircGLIS3(2)*

*CircGLIS3(2)* (circBase ID: hsa_circ_0 0 02874 and mmu_circ_0 0 07673) is a conserved circRNA which derives from the second exon of the *GLIS family zinc finger 3 (GLIS3)* gene in both human and mouse (**Figure** **2**A).^[^ ^8^, ^9^ ^]^ Its circular nature was confirmed through RT‐PCR analysis, which amplified the BSJ region absent in its linear form. This was further supported by its resistance to RNase R digestion, which typically degrades linear RNAs (Figure 2B,C).^[^ ^10^ ^]^ The predicted BSJ regions were verified by sequencing the PCR products (Figure 2D).

We then examined the subcellular location of *CircGLIS3(2)* by separating human primary fibroblasts into nucleus, cytoplasm, and mitochondria fractions. These fractions were enriched with *MALAT1* (a nuclear long non‐coding RNA),^[^ ^11^ ^]^ *GAPDH* mRNA (cytoplasmic), and *16S* rRNA (mitochondrial), respectively.^[^ ^12^ ^]^ We discovered that *CircGLIS3(2)* is primarily located in the cytoplasm of fibroblasts (Figure 2E), confirmed by in situ hybridization analysis (Figure 2F,G; Figure S1A, Supporting Information).

We also assessed the stability of *CircGLIS3(2)* in human dermal fibroblasts by inhibiting RNA transcription with Actinomycin‐D (5 µg mL^−1^).^[^ ^13^ ^]^ qRT‐PCR analysis revealed that *CircGLIS3(2)* has a half‐life of 22 h, which is considerably longer than the 8‐hour half‐life of linear *GLIS3* mRNA or the 18 h half‐life of *ACTB* mRNA (Figure 2H).

To investigate the increase in *CircGLIS3(2)* expression after skin injury, we treated human dermal fibroblasts with cytokines and growth factors relevant to wound repair (Figure 2I). IL‐1α elevated *CircGLIS3(2)* levels 24 h post‐treatment (Figure 2J), whereas its linear form increased as early as 2 h post‐treatment (Figure S1B, Supporting Information). This delayed induction of *CircGLIS3(2)* was further confirmed by analyzing nascent RNA in fibroblasts (Figure S1C,D, Supporting Information). Blocking IL‐1α downstream signaling with p38 inhibitors—alone or in combination with JNK or ERK inhibitors—abolished IL‐1α‐induced *CircGLIS3(2)* expression in human dermal fibroblasts (Figure S1E, Supporting Information). Additionally, we observed a significant positive correlation between IL‐1α and *CircGLIS3(2)* levels in human skin and wounds (Figure 2K). As IL‐1α is an early signal at wound sites,^[^ ^14^ ^]^ our findings suggest that injury‐induced IL‐1α rapidly upregulates *GLIS3* gene transcription in wound fibroblasts, while *CircGLIS3(2)* production requires more time than its linear counterpart.

### 2.3 Cap‐Independent Translation of *CircGLIS3(2)*

**Figure 3:**
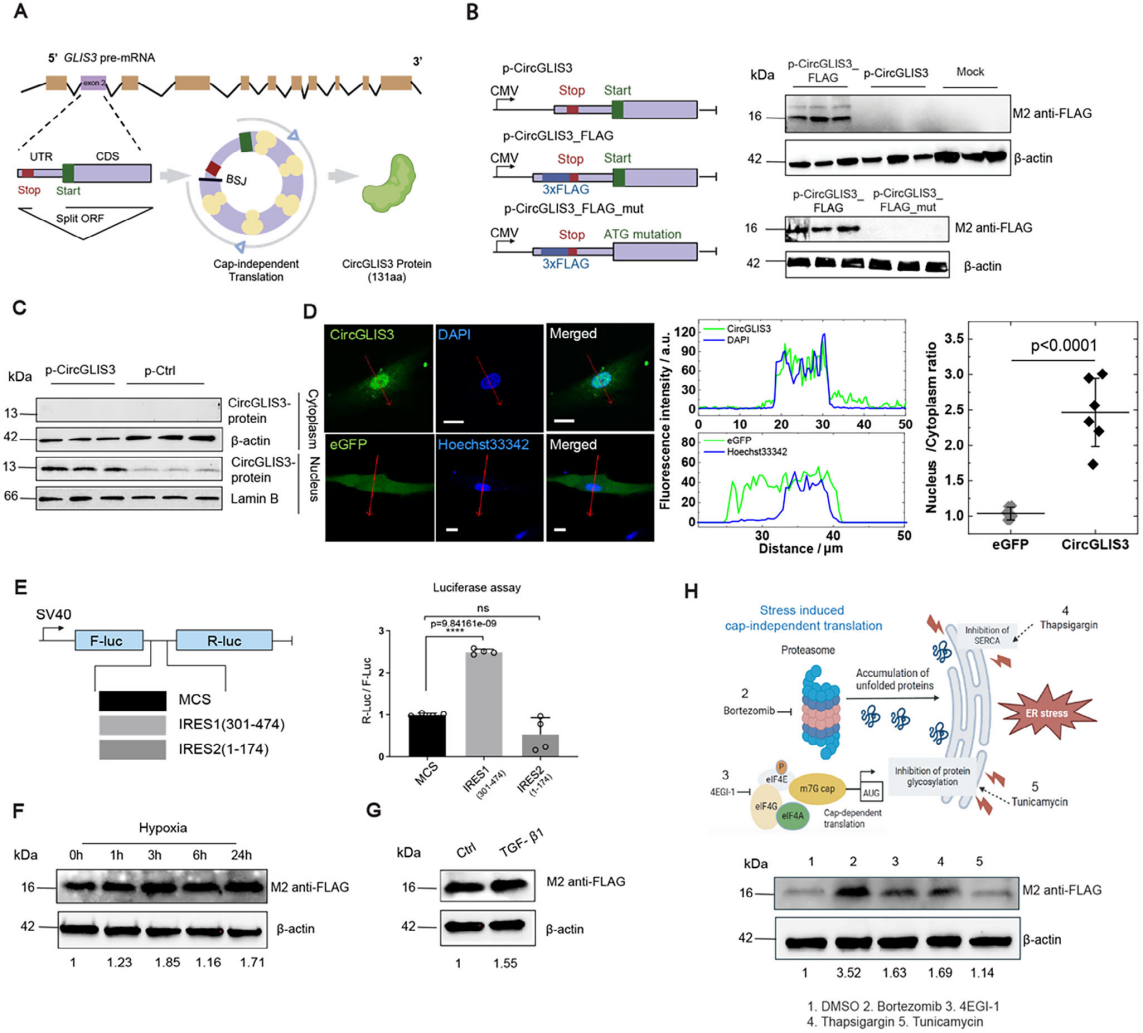
Cap‐independent translation of *CircGLIS3(2)*. A) Illustration of CircGLIS3(2) protein biogenesis. UTR: Untranslated Region; CDS: Coding Sequence; ORF: Open Reading Frame. B) Western blot analysis of FLAG expression in HEK293 cells transfected with p‐CircGLIS3, p‐CircGLIS3_FLAG, or p‐CircGLIS3_FLAG_mut. C) Western blot analysis of CircGLIS3(2) protein in human dermal fibroblasts transfected with p‐CircGLIS3 or control vectors for 24 h and then being separated into cytoplasmic (top) and nuclear (bottom) fractions. D) Immunofluorescence (IF) staining of CircGLIS3‐FLAG protein in fibroblasts transfected with p‐CircGLIS3_FLAG. Left panel: confocal laser scanning fluorescence microscopy image of fibroblasts expressing CircGLIS3(2)‐FLAG protein (top row) or eGFP (bottom row) as a homogenously distributed control, and with nuclear markers, DAPI or Hoechst33342. Red arrows indicate line profile position. Scale bar: 20 µm. Middle panel: line profile of fluorescence intensity along with the red arrows. Green: CircGLIS3(2)‐FLAG protein or eGFP, Blue: DAPI or Hoechst33342. Right panel: fluorescence intensity ratio of the nucleus to the cytoplasm in randomly selected cells (n = 6). E) Schematic representation of the luciferase bicistronic constructs used. The SV40 promoter drives the transcription of Firefly (F‐Luc) and Renilla (R‐Luc) luciferase‐coding regions in the control construct (MCS). The spacer was substituted with the predicted IRES sequences of *CircGLIS3(2)*. Luciferase activity derived from HEK293 cells transfected with these constructs are expressed as ratio of Renilla versus Firefly activities. Western blot analysis of CircGLIS3‐FLAG protein in HEK293 cells under hypoxia condition F), treated with TGF‐β1 G), or chemicals that induce ER stress H). Data are presented as means ± SD. ns P ≥ 0.05 and ****P<0.0001by Student's t‐test (D, E).

Upon examining the sequence of *CircGLIS3(2)* using data available in the circbank database (http://www.circbank.cn/), we identified a 396‐nucleotide open reading frame (ORF) within this circRNA, spanning from the putative start codon (ATG) of the host gene to a stop codon (TAA) created 8 nucleotides (nt) after the BSJ (**Figure** **3**A; Figure S2A, Supporting Information).^[^ ^15^ ^]^

To assess *CircGLIS3(2)*’s protein‐coding potential, we cloned its cDNA into the pLC5‐ciR vector, enabling circular transcript production. This resulted in the p‐CircGLIS3 plasmid, which expressed high levels of *CircGLIS3(2)* without affecting GLIS3 mRNA expression (Figure S2B,C, Supporting Information). We further modified this plasmid to create p‐CircGLIS3_FLAG, adding a 3×FLAG sequence just before the stop codon to tag the resulting protein (Figure 3B). Western blot analysis detected a 16 kDa flagged protein in HEK293 cells transfected with p‐CircGLIS3_FLAG, but not in mock‐treated cells or those transfected with p‐CircGLIS3 alone (Figure 3B). Mutating the start codon (ATG to TTT, p‐CircGLIS3_FLAG_mut) abolished protein production, confirming the translation initiation at this site (Figure 3B). These findings suggest that *CircGLIS3(2)* encodes a 131‐amino‐acid protein, overlapping with the N‐terminal region of the GLIS3 protein, with a single amino acid difference at the 3′ end (Figure S2D, Supporting Information). An antibody targeting the novel CircGLIS3(2) protein confirmed its presence in human dermal fibroblasts, with increased expression upon *CircGLIS3(2)* overexpression, as shown by Western blotting (Figure 3C). Notably, the CircGLIS3(2) protein was localized specifically in the cell nucleus (Figure 3C), confirmed by immunofluorescence (IF) analysis of the flagged version in p‐CircGLIS3_FLAG‐transfected human fibroblasts (Figure 3D; Figure S2E, Supporting Information).

Cap‐independent translation of circRNAs is often driven by IRES.^[^ ^6^ ^]^ We predicted two IRESs in the *CircGLIS3(2)* sequence (Figure S2A, Supporting Information) and subcloned each into a bicistronic construct between the Firefly and Renilla luciferase genes (Figure 3E). Compared to the control vector, the construct with IRES 1 displayed significantly higher Renilla luciferase activity, indicating that IRES 1 is crucial for *CircGLIS3(2)* translation (Figure 3E). Additionally, m6A modifications can enhance cap‐independent translation of circRNAs, either alone or alongside IRES elements.^[^ ^6^ ^]^ Using the sequence‐based RNA adenosine methylation site predictor (SRAMP) tool, we predicted an m6A site at position 33 with high confidence (Figure S2F, Supporting Information).^[^ ^16^ ^]^ In line with this, the expression of EIF4G2 and YTHDF3, which can recognize m6A and initiate translation, was found to be upregulated during human skin wound healing, potentially facilitating *CircGLIS3(2)* translation (Figure S2G,H, Supporting Information).^[^ ^6^, ^17^ ^]^

Cells often use cap‐independent translation to rapidly respond to stress stimuli, enabling the selective translation of specific RNAs.^[^ ^6^ ^]^ We found that heat shock, hypoxia, and TGF‐β1 treatment enhanced *CircGLIS3(2)* translation in HEK293 cells transfected with p‐CircGLIS3_FLAG (Figure 3F,G; Figure S2I, Supporting Information). Notably, Asiaticoside, which suppresses TGF‐β/Smad signaling by inducing Smad7 and inhibiting TGF‐βRI and TGF‐βRII,^[^ ^18^ ^]^ reduced TGF‐β‐induced *CircGLIS3(2)* translation (Figure S2J, Supporting Information). Additionally, several endoplasmic reticulum (ER) stress inducers—including Bortezomib, 4EGI‐1, thapsigargin, and tunicamycin—further boosted *CircGLIS3(2)* translation (Figure 3H). These compounds induce ER stress by disrupting protein degradation or folding, which triggers the unfolded protein response (UPR) and subsequent cellular stress mechanisms.^[^ ^19^, ^20^, ^21^, ^22^ ^]^ Hypoxia, TGF‐β signaling, and ER stress are known to play critical roles in wound repair.^[^ ^2^, ^23^ ^]^ Consistent with this, we observed increased expression of ER stress‐related genes, such as HSPA5, EDEM1, and ATF4, in dermal fibroblasts from human acute wounds, as revealed by single‐cell RNA sequencing (Figure S2K, Supporting Information).^[^ ^24^, ^25^, ^26^, ^27^ ^]^ These findings suggest that skin injury promotes *CircGLIS3(2)* translation, leading to the production of a novel *CircGLIS3(2)*‐derived protein isoform localized in the nucleus of wound fibroblasts.

### 2.4 Divergent Functions of *CircGLIS3(2)* RNA and Protein in Wound Fibroblasts

To investigate *CircGLIS3(2)*’s cellular functions, we manipulated its expression in human dermal fibroblasts. Three siRNAs were designed to specifically target *CircGLIS3(2)*’s BSJ, with two controls: one siRNA with partial (10/21 nucleotides) sequence complementarity to the BSJ (si‐CircGLIS3‐ctrl), and another scrambled sequence (si‐ctrl, Figure S3A, Supporting Information). Silencing efficiency was confirmed by qRT‐PCR, with no effect on linear *GLIS3* mRNA levels (Figure S3B,C, Supporting Information). For further experiments, we used si‐CircGLIS3_3. To overexpress *CircGLIS3(2)*, we employed p‐CircGLIS3, which increased both *CircGLIS3(2)* RNA and protein without affecting *GLIS3* mRNA (Figure 3C; Figure S2B,C, Supporting Information).

**Figure 4:**
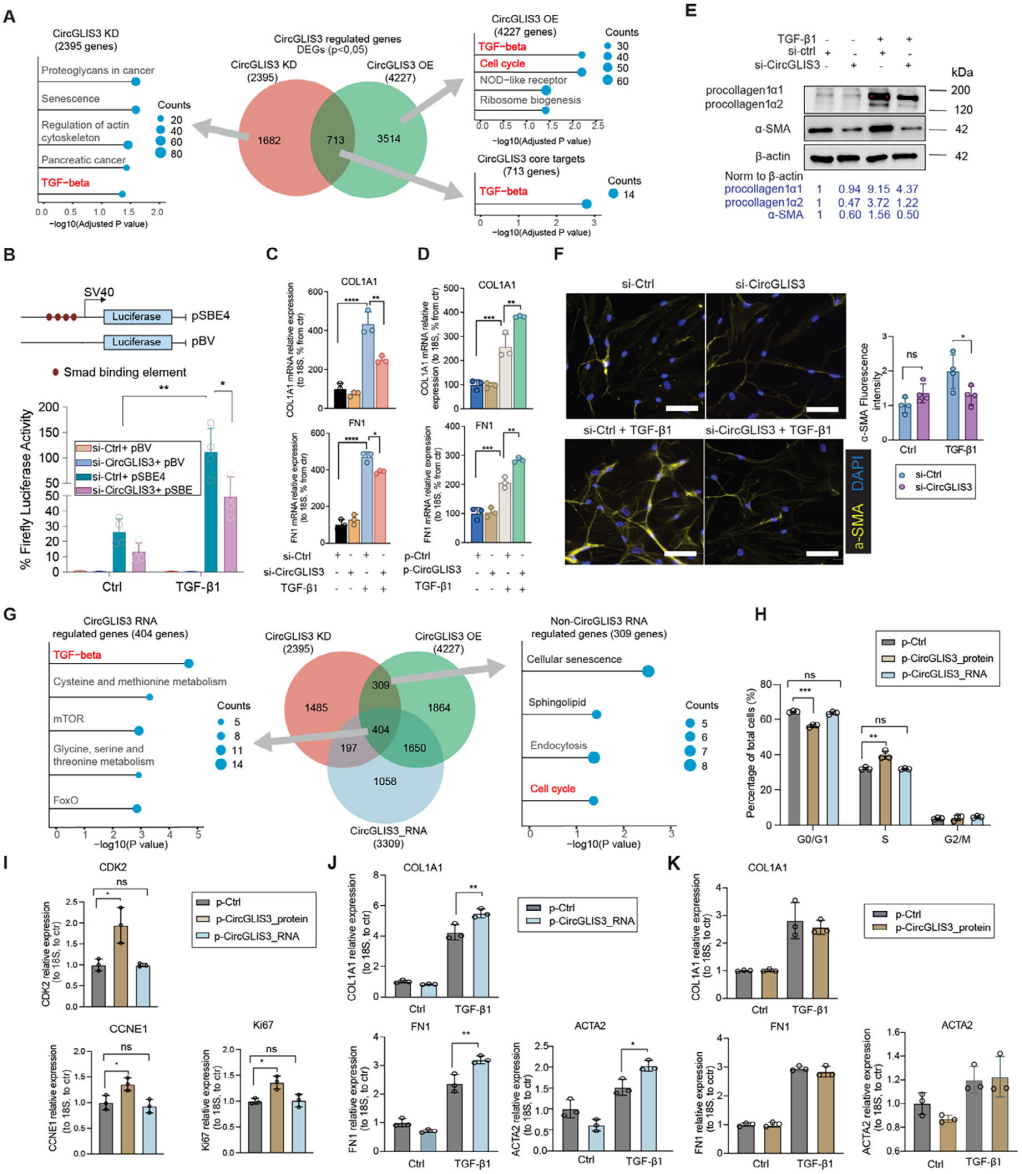
Divergent functions of *CircGLIS3(2)* RNA and protein in wound fibroblasts. A) Microarray analysis of human fibroblasts with *CircGLIS3(2)* overexpression (OE) or silencing (KD). KEGG enrichment analysis was performed for the differentially expressed genes (DEGs). B) Luciferase activity was measured in fibroblasts transfected with a TGF‐β reporter vector (pSBE4) or an empty vector (pBV), along with either si‐Ctrl or si‐CircGLIS3, for 24 h, followed by TGF‐β1 treatment for another 24 h (n = 4). qRT‐PCR of *COL1A1* and *FN1* mRNA in fibroblasts transfected with si‐Ctrl or si‐CircGLIS3 C), and in fibroblasts transfected p‐CircGLIS3 or p‐Ctrl vector for 24 h, followed by TGF‐β1 treatment for another 24 h (n = 4). D). E) Western blot of procollagen type 1 and α‐SMA in fibroblasts with *CircGLIS3(2)* KD and TGF‐β1 treatment. F) Immunofluorescence staining of α‐SMA in fibroblasts with *CircGLIS3(2)* KD and TGF‐β1 treatment. Scale bar = 100 µm. The signal intensity was quantified. G) A Venn diagram compared DEGs in human fibroblasts with *CircGLIS3(2)* OE, KD, or *CircGLIS3(2)*_RNA OE. KEGG enrichment analysis was conducted on the DEGs. H) Cell cycle analysis of human dermal fibroblasts transfected with p‐CircGLIS3_protein or p‐CircGLIS3_RNA or p‐Ctrl vector. I) qRT‐PCR analysis of cell cycle marker genes CDK2, CCNE1 and Ki67 in fibroblasts with *CircGLIS3(2)* RNA or protein OE. qRT‐PCR of *COL1A1, FN1*, and *ACTA2* mRNA in fibroblasts transfected with p‐CircGLIS3_protein J), or p‐CircGLIS3_RNA vectors for 24 h K) and then stimulated with TGF‐β1 for another 24 h (n = 3). Data are presented as means ± SD. ns P ≥ 0.05, *P<0.05 **P<0.01, ***P<0.001, and ****P<0.0001 by Two‐way ANOVA and Multiple comparisons (B) or one way ANOVA and Tukey's multiple comparisons test (C, D, J, K) or Student's t‐test (F, H, I).

Next, we performed transcriptomic profiling using microarrays on fibroblasts with either overexpression or silencing of *CircGLIS3(2)*. This revealed thousands of differentially expressed genes (DEGs), with 713 common to both conditions, suggesting they are core targets of *CircGLIS3(2)* (**Figure** **4**A; Figure S4A,B and Table S4, Supporting Information). KEGG enrichment analysis showed that these DEGs are involved in critical wound healing processes, especially TGF‐β signaling pathways, with *CircGLIS3(2)* overexpression also affecting the cell cycle (Figure 4A). Gene Set Enrichment Analysis (GSEA) further revealed that TGF‐β pathway genes, from the GSE79621 dataset,^[^ ^28^ ^]^ were enriched among those downregulated upon *CircGLIS3(2)* silencing, suggesting that *CircGLIS3(2)* is required for the TGF‐β signaling (Figure S4E, Supporting Information).

TGF‐β1 is a key growth factor that drives fibroblast‐to‐myofibroblast transformation and ECM remodeling during wound repair.^[^ ^29^, ^30^ ^]^ To investigate whether *CircGLIS3(2)* influences TGF‐β pathway activity, we co‐transfected human dermal fibroblasts with si‐CircGLIS3 and a luciferase reporter construct containing Smad‐binding elements (pSBE4).^[^ ^31^ ^]^ Our results showed that TGF‐β1 treatment increased luciferase activity, but this effect was significantly reduced when *CircGLIS3(2)* was silenced, indicating *CircGLIS3(2)*’s crucial role in TGF‐β1 signaling (Figure 4B). Consistent with this, we observed that *CircGLIS3(2)* knockdown led to a significant downregulation of several TGF‐β1‐induced target genes, including matrisome genes *FN1* and *COL1A1*, as well as contractility‐related gene *ACTA2* that encodes α‐SMA protein (Figure 4C; Figure S4F, Supporting Information). Conversely, overexpression of *CircGLIS3(2)* further enhanced their expression (Figure 4D; Figure S4G, Supporting Information). Furthermore, *CircGLIS3(2)* silencing reduced TGF‐β1‐induced procollagen type 1 and α‐SMA protein levels, as confirmed by Western blotting (Figure 4E) and IF analysis (Figure 4F).

To further dissect the functions of *CircGLIS3(2)* RNA and protein, we created two constructs: p‐CircGLIS3_RNA, expressing only *CircGLIS3(2)* RNA by mutating the start codon of *CircGLIS3(2)* translation in p‐CircGLIS3, and p‐CircGLIS3_protein, expressing only the CircGLIS3(2) protein without the circular RNA (Figure S3D–G, Supporting Information). Microarray analysis of fibroblasts overexpressing *CircGLIS3(2)* RNA revealed 3309 DEGs (p<0.05) (Figure S4C, Supporting Information), with enrichment in the TGF‐β signaling pathway (Figure S4D, Supporting Information). Among the 713 core targets of *CircGLIS3(2)* (Figure 4A), 404 were regulated by *CircGLIS3(2)* RNA and were enriched in the TGF‐β signaling pathway, while the remaining 309 core targets, unaffected by *CircGLIS3(2)* RNA, were enriched in cell cycle‐related genes (Figure 4G and Table S4, Supporting Information). Consistent with this, CircGLIS3(2) protein, but not RNA, promoted fibroblast progression from G1 to S phase, and upregulated cell proliferation and G1/S transition markers, including CDK2, CCNE1, and Ki67 (Figure 4H,I; Figure S4H, Supporting Information). Conversely, *CircGLIS3(2)* RNA, but not protein, enhanced TGF‐β1‐induced expression of ECM genes (*COL1A1, FN1*) and ACTA2 (Figure 4J,K).

In summary, our findings reveal that *CircGLIS3(2)* RNA and protein have distinct roles in dermal fibroblasts: its RNA promotes TGF‐β1‐induced activation into matrix‐secreting fibroblasts, while its protein stimulates fibroblast proliferation. Together, these functions enhance fibroblast activity during wound repair.

### 2.5 *CircGLIS3(2)* RNA Interacts with and Stabilizes PCOLCE Protein

**Figure 5:**
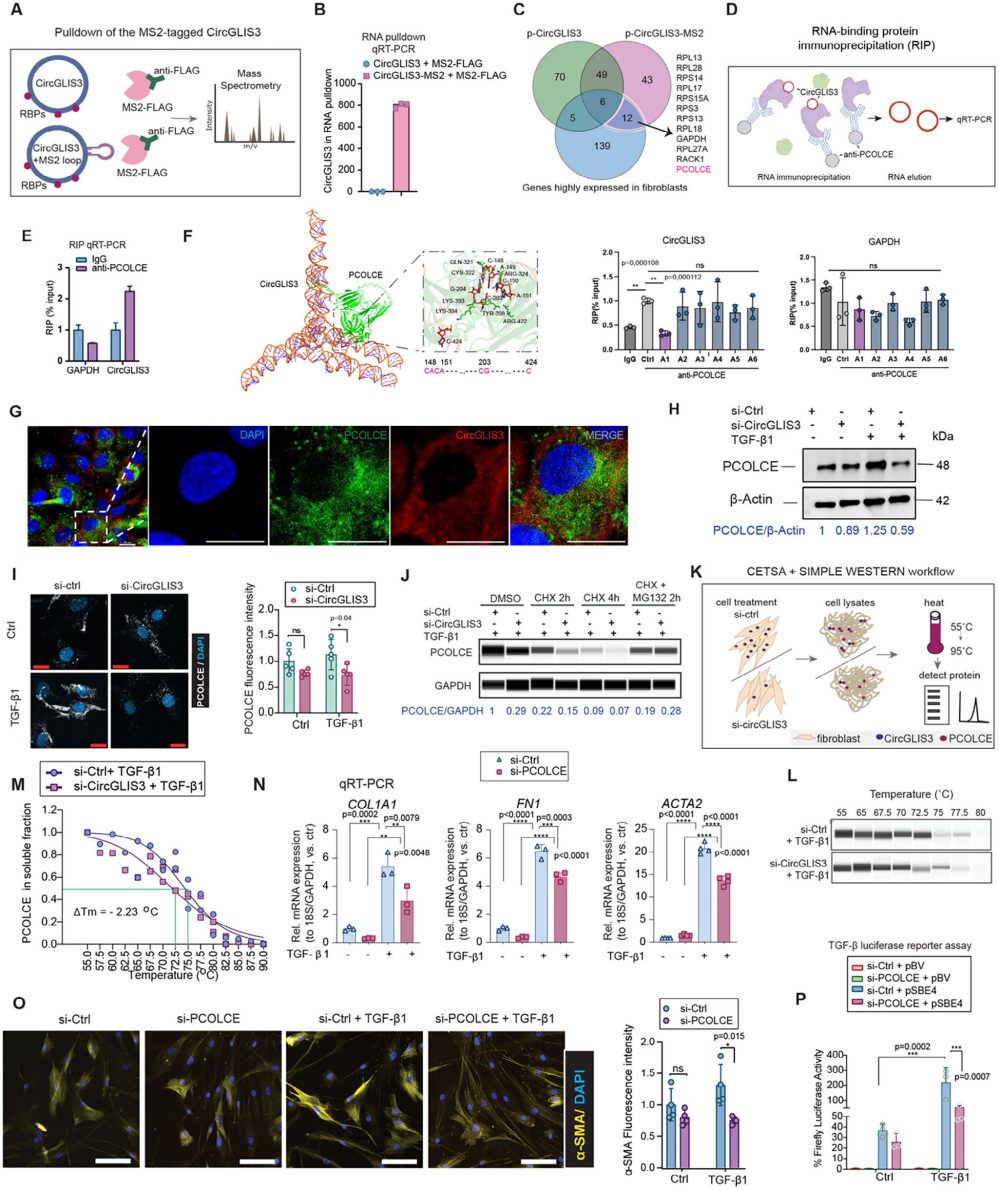
*CircGLIS3(2)* RNA interacts with and stabilizes PCOLCE protein. A) A vector containing CircGLIS3(2) tagged with MS2 hairpins (CircGLIS3‐MS2) was co‐transfected with a vector expressing a FLAG‐tagged fusion protein with an MS2‐recognizing portion (MS2‐FLAG) in HEK293 cells. Control cells were co‐transfected with CircGLIS3 plasmids and MS2‐FLAG. The ribonucleoprotein (RNP) complexes were isolated by using anti‐FLAG antibodies, and the eluted RNA‐binding proteins (RBP) were analyzed by mass spectrometry (MS). B) qRT‐PCR of *CircGLIS3(2)* in the RNPs. C) Venn diagram showing proteins identified by MS overlapped with a list of genes highly expressed in human dermal fibroblasts. D) Schematics of RNA‐binding protein immunoprecipitation (RIP). E) qRT‐PCR of *CircGLIS3(2)* and *GAPDH* mRNA in RNPs immunoprecipitated with anti‐PCOLCE antibody or IgG. F) Predicted structure of the *CircGLIS3(2)* RNA–PCOLCE complex with a zoomed‐in view of the binding interface (left panel). qRT‐PCR analysis of *CircGLIS3(2)* and *GAPDH* mRNA in RNPs immunoprecipitated using anti‐PCOLCE antibodies or IgG from fibroblasts transfected with antisense oligos (A1–A6) targeting potential PCOLCE binding sites in *CircGLIS3(2)* RNA (n = 3, right panel). G) Combined fluorescent in situ hybridization (FISH) and immunofluorescence (IF) analysis of *CircGLIS3(2)* RNA and PCOLCE protein in human dermal fibroblasts, with nuclei counterstained by DAPI. Scale bar: 20 µm. Western blotting H) and IF analysis I) of PCOLCE in fibroblasts with *CircGLIS3(2)* depletion and TGF‐β1 treatment. Scale bar = 20 µm. J) Simple Western analysis of PCOLCE in fibroblasts subjected to *CircGLIS3(2)* depletion and TGF‐β1 treatment for 24 h, followed by treatment with cycloheximide (CHX) and MG132 for 2 to 4 h. K) Illustration of CEllular Thermal Shift Assay (CETSA^®^) to compare cellular thermal stability of PCOLCE protein in the presence and absence of *CircGLIS3(2)*. Simple Western L) and melting curves M) of PCOLCE protein in human fibroblasts with *CircGLIS3(2)* silencing KD. N) qRT‐PCR of *COL1A1*, *FN1*, and *ACTA2* mRNA in fibroblasts with PCOLCE silencing and treated with TGF‐β1 for 24 h (n = 3‐4). O) IF analysis of α‐SMA in fibroblasts with PCOLCE depletion and TGF‐β1 treatment. Scale bar = 100 µm. Signal intensity was quantified. P) Luciferase activity was measured in fibroblasts transfected with a TGF‐β reporter vector (pSBE4) or an empty vector (pBV), along with either si‐Ctrl or si‐ PCOLCE, for 24 h, followed by TGF‐β1 treatment for another 24 h (n = 3). Data are presented as means ± SD. ns P ≥ 0.05, *P<0.05 **P<0.01, ***P<0.001, and ****P<0.0001 by one way ANOVA and Tukey's multiple comparisons test (N), or Student's t‐test (F, I, O), or Two‐way ANOVA and Multiple comparisons (P).

Given that *CircGLIS3(2)* operates as both an RNA in the cytoplasm and a protein in the nucleus, we further investigated the molecular mechanisms of its non‐coding and coding forms. We first profiled the proteins interacting with *CircGLIS3(2)* RNA using a pulldown assay in HEK293 cells. *CircGLIS3(2)* was tagged with MS2 hairpins and co‐expressed with a FLAG‐tagged MS2‐binding protein (MS2‐FLAG) (**Figure** **5**A). The *CircGLIS3(2)*‐protein complexes were isolated using FLAG antibody‐conjugated beads. The efficiency of this process was confirmed by comparing qRT‐PCR results for *CircGLIS3(2)* in MS2‐tagged versus non‐tagged pulldown products (Figure 5B).

Label‐free mass spectrometry (MS) identified 55 proteins that specifically interacted with CircGLIS3(2)‐MS2 (Figure 5C and Table S5, Supporting Information). Comparing these to the top expressed genes in human dermal fibroblasts (Table S6, Supporting Information),^[^ ^27^ ^]^ we found that Procollagen C‐Proteinase Enhancer 1 (PCOLCE) and several ribosomal proteins were highly enriched and specifically bound to *CircGLIS3(2)* RNA. The ribosomal protein interaction suggests *CircGLIS3(2)* RNA's translation potential. We focused on the interaction between *CircGLIS3(2)* RNA and PCOLCE protein and confirmed it using PCOLCE antibody‐conjugated beads in RNA‐binding protein immunoprecipitation (RIP) assays with human dermal fibroblasts (Figure 5D). *CircGLIS3(2)*, but not *GAPDH* mRNA, was enriched in the anti‐PCOLCE group compared to the IgG control (Figure 5E). Moreover, we used AlphaFold3 (https://alphafoldserver.com)^[^ ^32^ ^]^ to predict the 3D structure of the *CircGLIS3(2)* RNA–PCOLCE complex and analyzed potential binding regions within *CircGLIS3(2)* using PyMOL^[^ ^33^ ^]^ (Figure 5F). To validate these in silico predictions, we designed six antisense oligos (ASOs, sequences and target regions in Table S8, Supporting Information) targeting the predicted PCOLCE binding sites and transfected them into human dermal fibroblasts (Figure S2A, Supporting Information). RIP assays showed that PCOLCE could not pull down *CircGLIS3(2)* RNA when ASO 1, which blocks positions 148–151 relative to the BSJ, was used. This suggests that this region is critical for PCOLCE binding (Figure 5F). Additionally, colocalization of *CircGLIS3(2)* RNA and PCOLCE protein in the cytoplasm of human dermal fibroblasts, observed via combined fluorescent in situ hybridization (FISH) and IF analysis, further supports their interaction (Figure 5G).

PCOLCE is known for its TGF‐β1‐induced expression and its crucial role in collagen biosynthesis.^[^ ^34^ ^]^ Western blotting (Figure 5H) and IF analysis (Figure 5I) showed that PCOLCE protein levels decreased upon *CircGLIS3(2)* knockdown in fibroblasts treated with TGF‐β1. We hypothesized that *CircGLIS3(2)* might be needed to maintain PCOLCE protein levels by either enhancing its production or preventing its degradation. To test this, we examined the turnover of endogenous PCOLCE protein in TGF‐β1‐stimulated fibroblasts treated with cycloheximide (CHX), a protein translation inhibitor, and inhibitors of proteasomal and lysosomal degradation pathways^[^ ^35^ ^]^ (Figure S5A, Supporting Information). Blocking protein degradation, but not translation, equalized PCOLCE levels between *CircGLIS3(2)*‐depleted and control fibroblasts, suggesting *CircGLIS3(2)*’s role in stabilizing PCOLCE (Figure 5J). This was confirmed by a Cellular Thermal Shift Assay (CETSA), which showed that *CircGLIS3(2)* silencing caused PCOLCE protein to shift to a lower melting temperature (Figure 5K–M).^[^ ^36^ ^]^

We further investigated PCOLCE's role in TGF‐β1 signaling in fibroblasts. Similar to *CircGLIS3(2)*, PCOLCE knockdown reduced the TGF‐β1‐induced expression of matrisome genes, including *COL1A1* and *FN1*, as well as the contractility‐related gene *ACTA2*/α‐SMA (Figure 5N,O; Figure S5B, Supporting Information). Additionally, a TGF‐β1‐responsive luciferase reporter assay showed that PCOLCE depletion decreased luciferase activity in human primary fibroblasts (Figure 5P). Together, these results suggest that *CircGLIS3(2)* RNA interacts with and stabilizes PCOLCE protein, which is required for TGF‐β1 signaling in human dermal fibroblasts.

### 2.6 *CircGLIS3(2)* Encoded Protein Binds BTF3 in Cell Cycle Regulation

**Figure 6:**
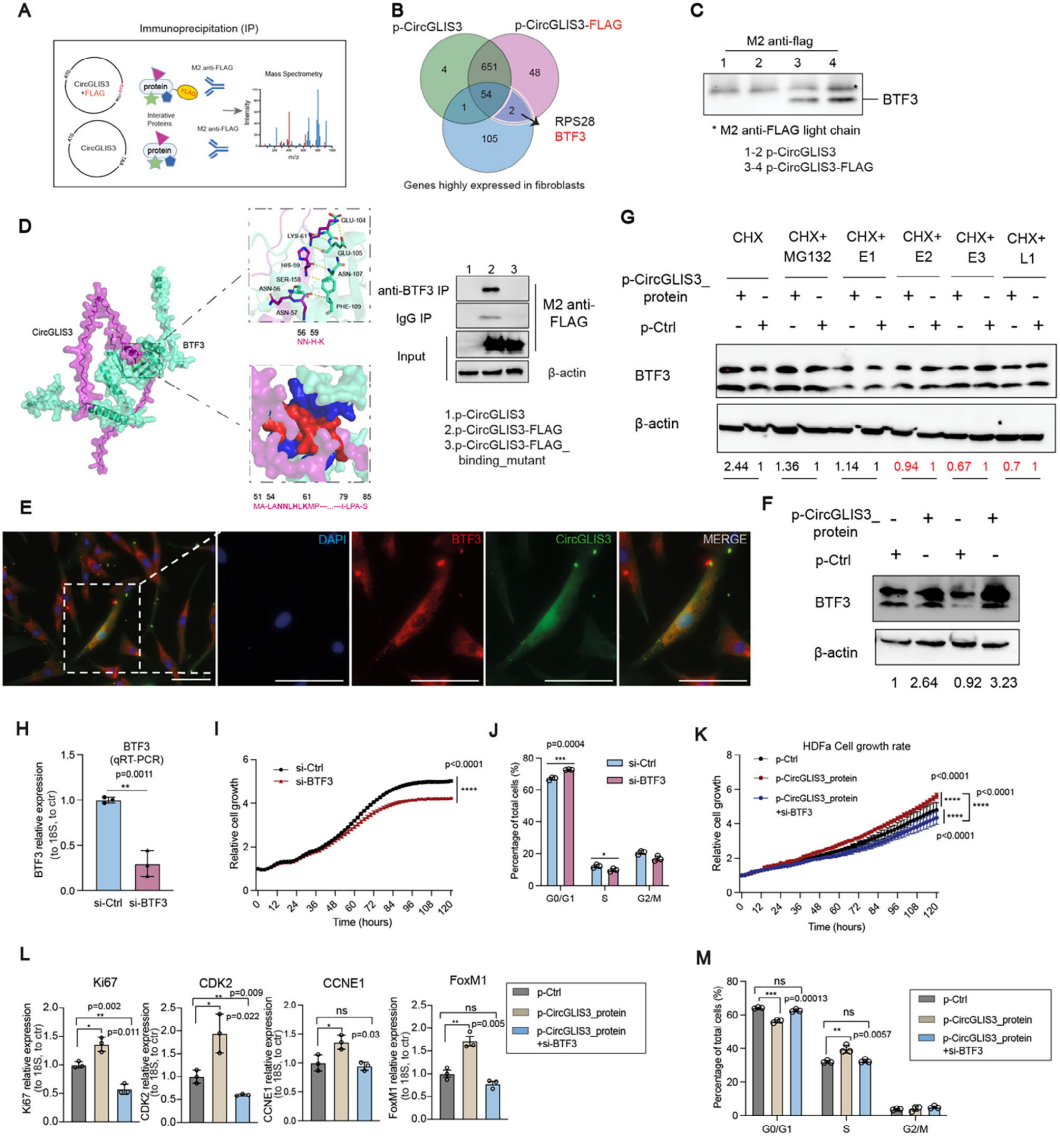
*CircGLIS3(2)* encoded protein binds BTF3 in cell cycle regulation. A) Illustration of CircGLIS3(2) protein immunoprecipitation (IP) in HEK293 cells transfected with p‐CircGLIS3_FLAG or p‐CircGLIS3 vectors. FLAG tagged CircGLIS3(2) protein and its binding partners were pull‐downed by using M2 anti‐FLAG antibody, and analyzed by mass spectrometry (MS). B) Venn diagram showing proteins identified by MS overlapped with a list of genes highly expressed in human dermal fibroblasts. C) Western blotting analysis of BTF3 in M2 anti‐FLAG IP. D) Predicted structure of the CircGLIS3(2)–BTF3 protein complex with a zoomed‐in view of the binding interface (left panel). Co‐IP assays in human dermal fibroblasts transfected with p‐CircGLIS3, p‐CircGLIS3‐FLAG, or p‐CircGLIS3‐FLAG with BTF3 binding site mutation (right panel). E) IF co‐staining of BTF3 (red) and CircGLIS3(2) proteins (green) in human dermal fibroblasts. Scale bar: 50 µm. F) Western blotting of BTF3 in fibroblasts with CircGLIS3(2) protein overexpression (OE). G) Western blotting of BTF3 in fibroblasts with CircGLIS3 protein OE and treated with cycloheximide (CHX) and MG132, or inhibitors for proteasome: E1, E2, E3 for proteasomal degradation, and L1 for lysosomal degradation. The levels of BTF3 relative to β‐actin were quantified. H) qRT‐PCR of BTF3 mRNA in human dermal fibroblasts transfected with BTF3 siRNA (n = 3). Cell growth (n = 3) I) and cell cycle analysis (n = 3) J) were performed in fibroblasts with BTF3 silencing. Cell growth (n = 3‐4) K), qRT‐PCR analysis of proliferation regulators (n = 3) L), and cell cycle analysis (n = 3) (M) in human fibroblasts with CircGLIS3(2) protein OE, along with or not with BTF3 silencing. Data are presented as means ± SD. ns P ≥ 0.05, *P<0.05 **P<0.01, ***P<0.001, and ****P<0.0001 by Two‐way ANOVA and Multiple comparisons (I, K) or Student's t‐test (H, J, L, M).

Next, we examined the protein interactome of *CircGLIS3(2)* encoded protein using immunoprecipitation (IP). HEK293 cells were transfected with p‐CircGLIS3_FLAG or p‐CircGLIS3, and the FLAG‐tagged CircGLIS3 protein complex was isolated for MS analysis (**Figure** **6**A). MS identified 50 proteins that specifically bind to the CircGLIS3(2) protein, with Basic Transcription Factor 3 (BTF3) and RPS28 being the most abundant in human dermal fibroblasts (Figure 6B and Tables S6 and S7, Supporting Information). Co‐immunoprecipitation (co‐IP) using FLAG antibody confirmed the interaction between CircGLIS3(2) and BTF3 proteins (Figure 6C). Furthermore, we used AlphaFold3^[^ ^32^ ^]^ to predict the 3D structure of the CircGLIS3(2)–BTF3 protein complex (Figure 6D). PyMOL analysis identified a potential BTF3‐binding motif in CircGLIS3(2) protein, consisting of residues 56, 57, 59, and 61 (NN‐H‐K). Additionally, the PRODIGY server^[^ ^37^ ^]^ redicted two BTF3‐binding regions: residues 51, 52, and 54–63 (MA‐LANNLHLKMP) and residues 79, 81–83, and 85 (I‐LPA‐S) (Figure 6D; Figure S2D, Supporting Information). To validate these findings, we mutated the predicted BTF3‐binding sites in the p‐CircGLIS3‐FLAG plasmid. Co‐IP assays in human dermal fibroblasts transfected with p‐CircGLIS3, p‐CircGLIS3‐FLAG, or p‐CircGLIS3‐FLAG with BTF3 binding site mutation showed that BTF3 antibody failed to pull down the mutated CircGLIS3‐FLAG protein, confirming the importance of these sites (Figure 6D). Moreover, IF co‐staining of BTF3 and CircGLIS3(2) proteins demonstrated their colocalization, further supporting their interaction in human dermal fibroblasts (Figure 6E).

We observed that BTF3 levels increased under stress conditions such as heat shock, hypoxia, ER stress, and TGF‐β1 treatment, similar to CircGLIS3(2) protein (Figure S6A–D, Supporting Information). Overexpression of CircGLIS3(2) protein notably enhanced BTF3 protein levels, but not RNA levels, in fibroblasts (Figure 6F; Figure S6E, Supporting Information). Blocking protein degradation (by MG132, E1, E2, E3, and L1 inhibitors), but not translation (by CHX), equalized BTF3 protein levels between CircGLIS3(2) protein‐overexpressing and control fibroblasts, suggesting that *CircGLIS3(2)* encoded protein stabilizes BTF3 protein (Figure 6G; Figure S5A, Supporting Information).

BTF3 is an oncogenic transcription factor that promotes the growth of various cancer cells.^[^ ^38^ ^]^ We knocked down BTF3 in human dermal fibroblasts using siRNA (Figure 6H; Figure S6F, Supporting Information), which led to reduced cell growth, altered cell cycle progression, and decreased expression of proliferation regulators, including *Ki67, CDK2, CCNE1*, and FoxM1—a key transcription factor involved in cell proliferation and cell fate decision^[^ ^39^ ^]^ (Figure 6I,J; Figure S6G,H, Supporting Information). Consistent with BTF3's role in regulating FoxM1 in cancer cells,^[^ ^40^, ^41^ ^]^ we found that CircGLIS3(2) protein, but not its RNA, enhanced FoxM1 expression at both the RNA and protein levels (Figure S6I,J, Supporting Information). Silencing BTF3 eliminated the positive effects of CircGLIS3(2) protein on fibroblast growth, cell cycle progression, and expression of proliferation regulators, indicating that BTF3 is a key mediator of the pro‐proliferation effects of CircGLIS3(2) protein (Figure 6K–M; Figure S6K, Supporting Information). Overall, our findings suggest that CircGLIS3(2) protein interacts with and stabilizes BTF3 protein, which is crucial for regulating the cell cycle and growth of human dermal fibroblasts.

### 2.7 *CircGLIS3(2)* is Essential for Wound Repair

**Figure 7:**
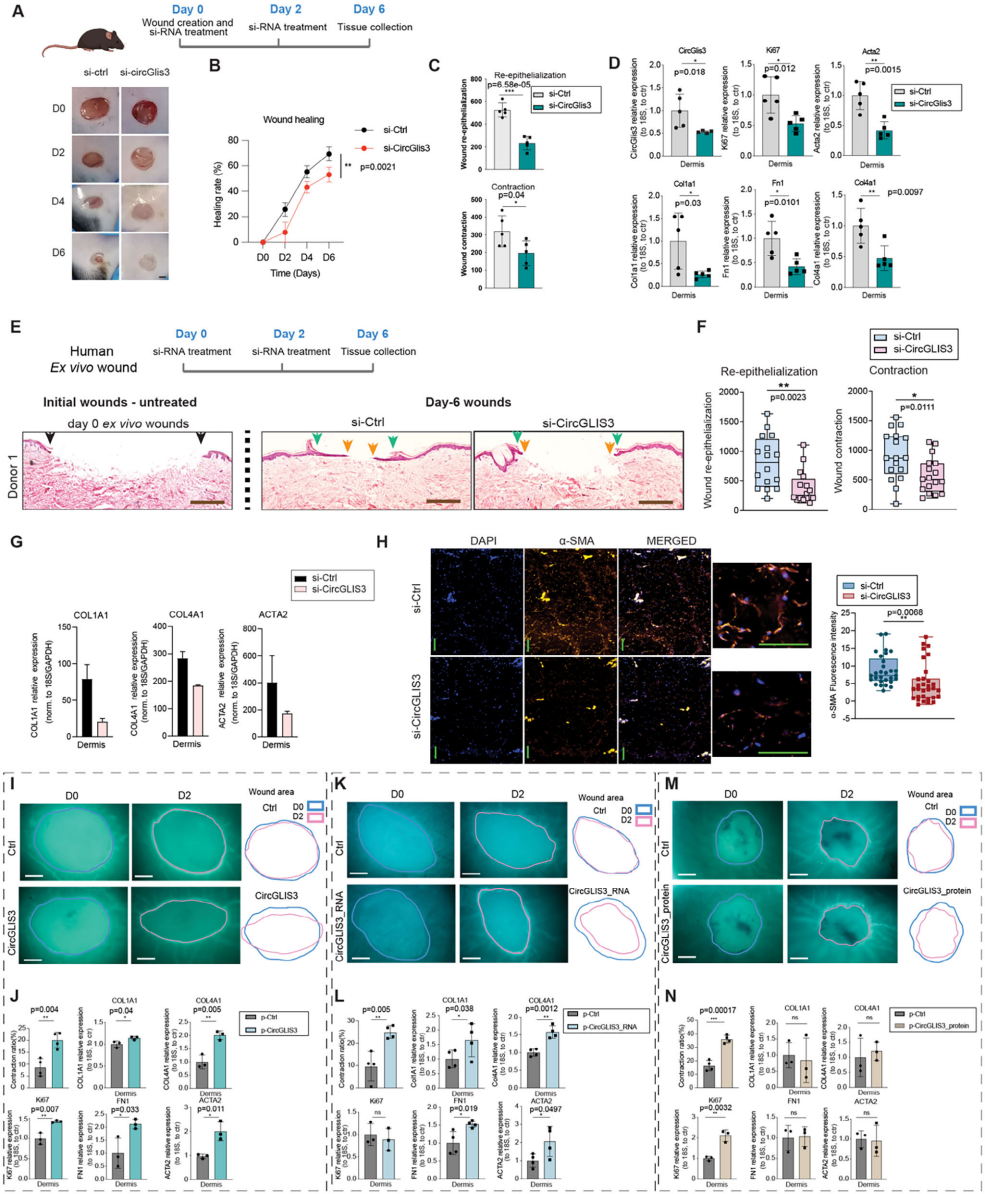
*CircGLIS3(2)* is essential for wound repair. A) Topical application of CircGlis3 or control siRNAs on mouse wounds (n = 12/group): representative wound images from Day‐0 (D0) to Day‐6 post‐injury (D6) and wound closure was quantified in B). C) Quantification of wound re‐epithelialization and contraction in hematoxylin and eosin (H&E) staining of D6 wound sections (n = 5 /group). D) qRT‐PCR analysis of *CircGlis3*, *Col1a1*, *Acta2*, *Fn1*, *Ki67* and *Col4a1* in the dermis of D6 wounds (n = 5). E) Human *ex vivo* wounds were treated with si‐CircGLIS3 or control siRNA. H&E staining was performed in day‐0 and day‐6 wounds. Black arrows demarcate the initial wound edges at day 0, green arrows indicate the wound edges at day 6, and orange arrows highlight the newly formed epidermis. Scale bar = 500 µm. F) Quantification of re‐epithelialization (distance between green arrows – distance between orange arrows) and contraction (distance between black arrows – distance between green arrows) of 3–4 wounds per donor for 3 donors. G) qRT‐PCR of *COL1A1*, *COL4A1* and *ACTA2* in the dermis of *ex vivo* wounds (n = 2 donors). H) Representative images of immunofluorescence (IF) analysis of α‐SMA in human *ex vivo* wounds (n = 6 /group). scale bar = 100 µm. The signal intensity was quantified. Human *ex vivo* wounds were treated with p‐CircGLIS3 I,J), or p‐CircGLIS3_RNA K,L), or p‐CircGLIS3_protein or control vectors (M, N) (n = 3‐4 /group). I,K,M) Representative images of *ex vivo* wounds on days 0–2 post‐injury (left panel). The wound edges were demarcated with dashed lines. The areas within the wound edge (IW_time point_) are illustrated (right panel). Scale bars: 500 µm. J,L,N) Wound contraction (%) was quantified as ΔIW_D2_ /IW_D0_×100% (n = 4 /group). qRT‐PCR of *COL1A1*, *COL4A1, Ki67, FN1*, and *ACTA2* in the dermis of *ex vivo* wounds (n = 3‐4) J). Data are presented as means ± SD. ns P ≥ 0.05, *P<0.05 **P<0.01, ***P<0.001, and ****P<0.0001 by Two‐way ANOVA analysis (B) or Student's t‐test (C, D, F, H, J, L, N).

Since *CircGLIS3(2)* is conserved in mice (circBase ID: mmu_circ_0 0 07673), we investigated its role in skin wound healing using a murine wound model (Figure 2A). Its expression gradually increased during wound repair, similar to human skin healing dynamics (Figure S7A, Supporting Information). Silencing *CircGlis3* in cultured mouse dermal fibroblasts reduced TGF‐β1‐induced expression of *Acta2*, *Col1a1*, and *Fn1* at both the mRNA and protein levels, mirroring the effects observed in human fibroblasts, suggesting a conserved function (Figure S7B,C, Supporting Information). Topical application of *CircGlis3* or control siRNAs on mouse wounds significantly delayed wound closure, as seen in both macroscopic and histological analyses (**Figure** **7**A–C; Figure S7D, Supporting Information). Wounds collected six days post‐injury from si‐*CircGlis3*‐treated mice showed reduced dermal expression of *CircGlis3, Ki‐67, Acta2*, and ECM genes (Figure 7D). siRNA treatment reduced *CircGlis3* levels specifically in the dermis, not the epidermis, highlighting the importance of dermal *CircGlis3* in wound repair (Figure S7E, Supporting Information).

Given the differences in skin anatomy and wound healing mechanisms between humans and mice,^[^ ^42^ ^]^ we assessed the role of *CircGLIS3(2)* in human *ex vivo* wounds to evaluate the translational value of our findings. Unlike murine models, the *ex vivo* model preserves key human‐specific features, including fibroblast behaviour, immune composition, and extracellular matrix architecture, providing a more physiologically relevant platform for wound healing studies (Figure S7F, Supporting Information).^[^ ^43^, ^44^ ^]^ We applied *CircGLIS3(2)* siRNAs or control oligos topically to human *ex vivo* wounds immediately after injury and again two days later. On Day 6, wounds treated with *CircGLIS3(2)* siRNA showed impaired closure, with significantly reduced re‐epithelialization and wound‐edge contraction, as seen in H&E staining (Figure 7E,F; Figure S7G, Supporting Information). qRT‐PCR confirmed effective reduction of *CircGLIS3(2)* levels in the dermis, but not the epidermis, by siRNA treatment (Figure S7H, Supporting Information). Consistent with our in vitro and in vivo findings, *CircGLIS3(2)* knockdown decreased the expression of contractility‐related gene *ACTA2* and matrisome genes *COL1A1* and *COL4A1* in human *ex vivo* wound dermis (Figure 7G). IF staining showed reduced α‐SMA expression in the dermal compartments of *CircGLIS3(2)*‐deficient wounds, explaining their lesser contraction (Figure 7H).

We also applied p‐CircGLIS3 with a transfection reagent topically on human *ex vivo* wounds, enhancing dermal *CircGLIS3(2)* levels (Figure S7I, Supporting Information). *CircGLIS3(2)* overexpression promoted wound contraction, increased the expression of *ACTA2*, matrisome genes (*COL1A1, FN1*, *COL4A1*), and proliferation marker *Ki67* (Figure 7I,J). Interestingly, both forms of *CircGLIS3(2)*—RNA and protein—promoted wound contraction, but with different effects: the RNA form mainly increased *ACTA2* and ECM gene expression in dermal cells, while the protein form primarily boosted cell proliferation, indicated by higher *Ki67* levels (Figure 7K–N; Figure S7I,J, Supporting Information). These findings were further confirmed at the protein level by IF analysis (Figure S8A–D, Supporting Information).

**Figure 8:**
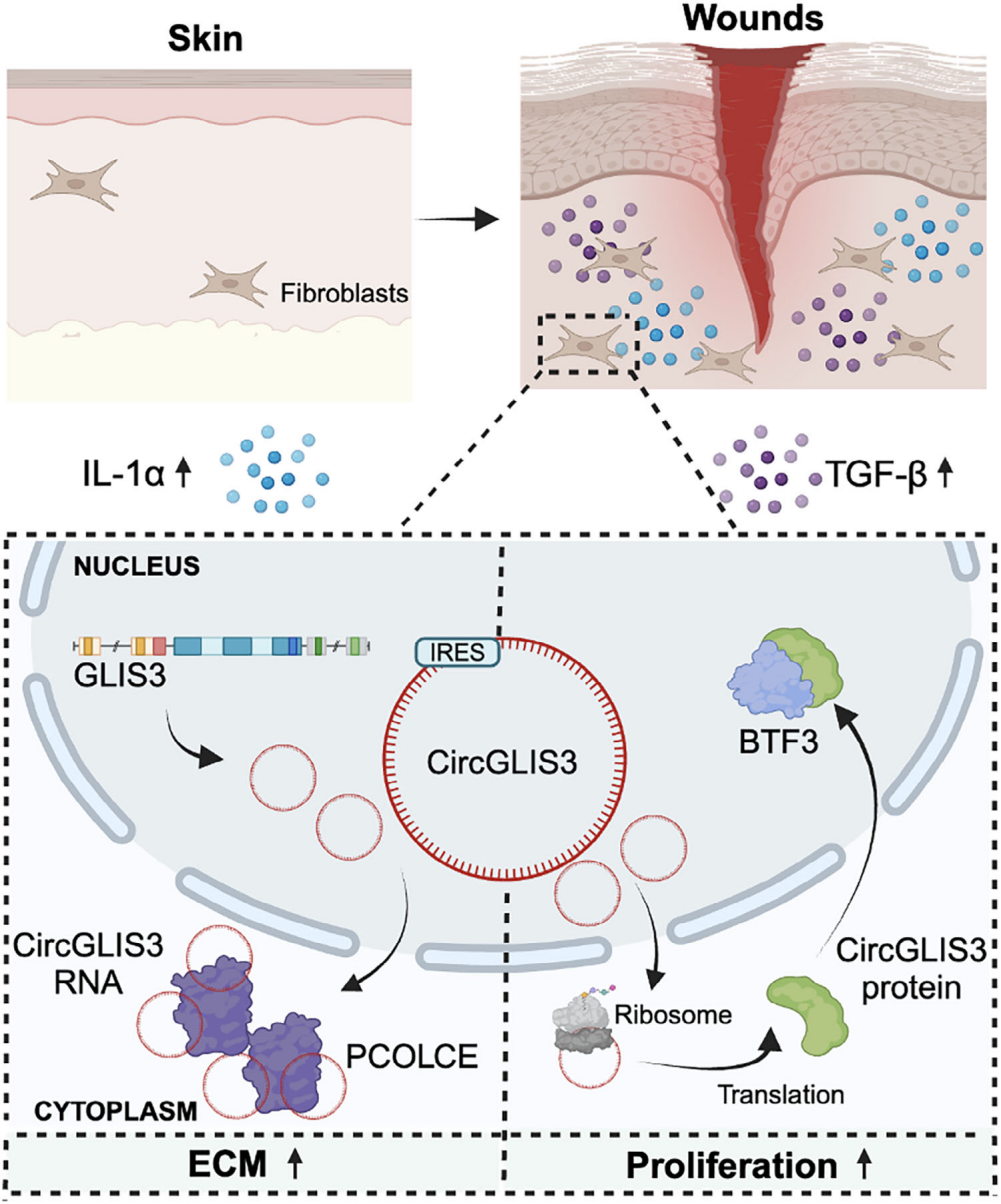
Schematic summary of the study. We identified *CircGLIS3(2)*, a circRNA transiently upregulated in dermal fibroblasts during human acute wound healing. Injury signals, such as IL‐1α and TGF‐β, induce its expression and translation. The RNA form activates fibroblasts into matrix‐secreting cells, while the encoded protein promotes cell proliferation, collectively enhancing wound repair. Mechanistically, *CircGLIS3(2)* RNA stabilizes cytoplasmic PCOLCE, while its protein interacts with nuclear BTF3. Both the RNA and protein are essential for wound closure.

These results reveal the novel bifunctional nature of *CircGLIS3(2)*: its RNA form promotes fibroblast activation into matrix‐secreting cells, while its protein form enhances fibroblast proliferation, collectively supporting wound repair (**Figure** **8**). Our findings underscore the versatile modes of action of a single gene under rapidly changing environmental and physiological conditions, which can be harnessed for disease management.

## 3 Discussion

Our study identifies *CircGLIS3(2)* as a significantly upregulated circular RNA in dermal fibroblasts during human skin wound healing. This circRNA has novel bimodal roles: its RNA form interacts with the cytoplasmic PCOLCE protein, enhancing fibroblast contraction and ECM production, while it also encodes a novel protein that stabilizes the nuclear transcription factor BTF3, promoting cell proliferation (Figure 8). These intertwined functions collectively facilitate wound repair.

Previous studies have shown that some translatable circRNAs function as non‐coding RNAs in one tissue while being translated in another, highlighting their cell‐ or tissue‐specific roles [reviewed in (*6*)]. For example, *circEgfr* acts as a tumor suppressor in glioma^[^ ^45^ ^]^ but has an oncogenic role in glioblastoma.^[^ ^46^ ^]^ Similarly, *circMapk1* RNA promotes vascular smooth muscle cell proliferation and migration,^[^ ^47^ ^]^ while its protein form inhibits gastric cancer.^[^ ^48^ ^]^ circZnf609 RNA functions as a tumor suppressor in colorectal cancer,^[^ ^49^ ^]^ whereas its protein form regulates myoblast proliferation in myogenesis.^[^ ^50^ ^]^ Unlike these examples, our study reveals the coexistence and cooperation of both coding and non‐coding functions of *CircGLIS3(2)* within the same cell type (fibroblasts) in a physiological context (wound healing). We propose that this dual function may not be unique to *CircGLIS3(2)* but could apply to other translatable circRNAs, which have often been studied only for their protein products. Our findings open new possibilities for exploring similar dual roles in other circRNAs across various biological contexts.

Dermal fibroblasts exist in different states during skin homeostasis and wound healing.^[^ ^51^ ^]^ In homeostatic skin, fibroblasts are mostly quiescent, synthesizing structural proteins and maintaining ECM balance. Upon wounding, they become activated, express α‐SMA, proliferate, migrate, and deposit ECM to reconstruct the wound bed.^[^ ^51^ ^]^ In wound fibroblasts, *CircGLIS3(2)* RNA expression is triggered by IL‐1α, while its translation is activated by TGF‐β and stress signals such as hypoxia—early responses to wounding absent in homeostatic skin.^[^ ^2^, ^23^ ^]^ The wound‐induced *CircGLIS3(2)* RNA and protein promote fibroblast activation and proliferation, respectively, which are crucial for wound repair. The transient and timely upregulation of *CircGLIS3(2)* is essential for proper healing. However, its persistent overexpression, as seen in keloids and hypertrophic scars,^[^ ^52^ ^]^ likely due to high TGF‐β and IL‐1α signaling,^[^ ^53^ ^]^ may contribute to dermal fibrosis, characterized by excessive α‐SMA‐positive (myo)fibroblasts, increased ECM deposition, and fibroblast hyperproliferation.^[^ ^54^, ^55^ ^]^

Cells use cap‐independent translation, such as IRES or m6A‐driven translation, for rapid and specific responses to stress, allowing immediate protein production crucial for stress response and cell cycle regulation.^[^ ^56^ ^]^ Tumor cells often exploit this strategy to produce oncogenic and survival‐promoting factors, gaining resistance to chemotherapy and radiotherapy.^[^ ^57^ ^]^ Notably, many translatable circRNAs are found in various cancers.^[^ ^6^ ^]^ *CircGLIS3(2)*, for instance, acts as an oncogenic factor in non‐small cell lung cancer, bladder cancer, and glioma.^[^ ^58^, ^59^, ^60^, ^61^ ^]^ It is also packaged into beta cell‐derived exosomes and transferred to islet endothelial cells, reducing angiogenesis and contributing to type 2 diabetes development.^[^ ^62^ ^]^ Further research is needed to determine whether the CircGLIS3(2) protein functions alongside its RNA form in these disease contexts.

*CircGLIS3(2)* uses both its RNA and protein forms to stabilize key elements in fibroblasts: PCOLCE in the cytoplasm and BTF3 in the nucleus. PCOLCE enhances collagen maturation by promoting bone morphogenetic protein 1/tolloid‐like proteinase activity, which cleaves C‐propeptides from procollagens—an essential step for collagen fibril formation.^[^ ^63^ ^]^ Our findings show that PCOLCE amplifies ECM production via TGF‐β1 signaling. BTF3, a transcription factor forming a stable complex with RNA polymerase IIB, is essential for transcription initiation and promotes fibroblast proliferation, consistent with its oncogenic role in various cancers.^[^ ^38^ ^]^ In line with increased *CircGLIS3(2)* expression in keloid tissue, both PCOLCE and BTF3 are implicated in skin fibrosis. Upregulated expression of PCOLCE, along with collagen type I and IV and fibronectin, are observed in hypertrophic and keloid scars.^[^ ^64^, ^65^ ^]^ BTF3 is also overexpressed in keloid fibroblasts, enhancing proliferation, migration, and activation of the JAK2/STAT3 pathway.^[^ ^66^ ^]^ Thus, through both non‐coding and coding mechanisms, *CircGLIS3(2)* regulates multiple aspects of fibrosis across subcellular compartments. This highlights the remarkable efficiency of the genome, using minimal genetic information to generate diverse products and support complex biological functions.

In summary, *CircGLIS3(2)* is a versatile regulator of fibroblast functions, contributing to both wound healing and pathological scarring. This makes *CircGLIS3(2)* a promising therapeutic target for improving wound healing and reducing excessive scarring. Our findings highlight how a circRNA can expand its functional range by utilizing both its RNA and protein forms, supporting cellular adaptability to environmental changes. Further research is needed to explore the broader implications of this phenomenon in RNAs with dual coding and non‐coding functions.

## 4 Experimental Section

### Study Design

The aims of this study were to i) identify circRNAs involved in human skin wound healing and ii) determine the physiological role of *CircGLIS3(2)* in wound fibroblasts, along with its molecular mechanism. RNA‐seq, LCM, and qRT‐PCR were used to identify and quantify circRNAs in skin and wound biopsies from healthy volunteers, and matched skin and lesion biopsies from keloid patients. Written informed consent was obtained from all donors. Samples from donors 1–35 were collected at Karolinska University Hospital Solna (Sweden), approved by the Stockholm Regional Ethics Committee. Keloid and matched skin samples from donors 36–43 were obtained from the Jiangsu Biobank of Clinical Resources (China), approved by the Ethics Committee of the Hospital for Skin Diseases, Chinese Academy of Medical Sciences and Peking Union Medical College. The study followed the principles of the Declaration of Helsinki. In vitro experiments assessed gene expression, cell function, and RNA‐protein or protein‐protein interactions in dermal fibroblasts isolated from human skin. A murine in vivo wound model and a human *ex vivo* wound model were used to evaluate the impact of *CircGLIS3(2)* on wound healing. Details on sample sizes, replicates, and statistical methods are provided in the figure legends and “Statistical analysis” section.

### Human Skin and Wound Specimens

To investigate circRNA expression during human skin wound healing, skin and wound biopsies were collected from 27 healthy volunteers (Tables S1 and S2, Supporting Information). Donors were excluded if they had diabetes, skin diseases, unstable heart conditions, infections, bleeding disorders, immune suppression, or ongoing medical treatments. Each donor received two or three 4‐mm excisional wounds, and the excised skin served as an intact skin control. Wound‐edge tissues were collected using a 6‐mm punch at 1, 7, and 30 days post‐wounding. Anesthesia was administered via local lidocaine injection. For donors 1–10, full‐thickness wound‐edge tissues were used for RNA‐seq and qRT‐PCR; for donors 11–20, for magnetic‐activated cell sorting (MACS); and for donors 21–27, for laser capture microdissection (LCM) (Table S2, Supporting Information). Additionally, skin discarded from plastic surgeries was collected to establish an *ex vivo* wound model (donors 28–33) and isolate dermal fibroblasts (donors 34–35) (Table S2, Supporting Information). Keloid and adjacent normal skin tissues were collected during surgeries from donors 36–43 (Table S2, Supporting Information).

### RNA Sequencing

Total RNA was isolated from full‐thickness skin biopsies, Wound1, and Wound7 (n = 5 per group), as well as from isolated keratinocytes and fibroblasts from skin and Wound7 (n = 5 per group) (Tables S1 and S2, Supporting Information) using the miRNeasy Mini Kit (Qiagen). Ribosomal RNA was removed using the Epicentre Ribo‐zero® rRNA Removal Kit, with 2 µg total RNA as input for each library. Strand‐specific RNA‐seq libraries were then prepared using the NEBNext® Ultra™ Directional RNA Library Prep Kit for Illumina®, according to the manufacturer's instructions. RNA‐seq libraries for isolated keratinocytes and fibroblasts were constructed following the NuGen Ovation Solo RNA‐Seq System tutorial. Libraries were sequenced on the Illumina Hiseq 4000 platform with 150 bp paired‐end reads.

### MS2‐Mediated Pulldown of *CircGLIS3(2)*‐Bound Proteins and Mass Spectrometry

Pulldown of MS2‐tagged *CircGLIS3(2)* and its protein interactome was performed using previously published methods.^[^ ^67^, ^68^ ^]^ In brief, it was co‐transfected HEK293T cells with 1 µg CircGLIS3 overexpression plasmids with or without the MS2 hairpins (p‐CircGLIS3 and p‐CircGLIS3‐MS2) together with a captured protein expression plasmid (MS2‐CP) containing a FLAG tag for 48 h by using Lipofectamine™ Reagent and PLUS™ reagent (ThermoFisher Scientific) (Figure 4A). The immunoprecipitation of the CircGLIS3‐RBP complex was performed with protein A+G beads coated with an anti‐FLAG antibody. RNA‐protein complexes were eluted from the beads; RNA and protein fractions were isolated. The enrichment of *CircGLIS3(2)* in the CircGLIS3‐MS2 group after immunoprecipitation was validated by qRT‐PCR. The protein fractions from CircGLIS3‐MS2 (test) and CircGLIS3 (control) were analyzed with mass spectrometry. Briefly, the proteins in the eluate were reduced with 0.05 M TCEP solution at 60 °C for 1 h and then alkylated with 55 nM MMTS for 45 min at room temperature. The solution was filtered on 10 kDa centrifugal filter devices for 20 min at 12 000 x *g*. The proteins were then digested with trypsin at 37 °C overnight using an enzyme‐to‐protein ratio of 1:50. The resulting peptides were collected by centrifugation and vacuum dried at low temperature. Peptides were then dissolved in 2% ACN and 0.1% formic acid and analyzed on a Thermo Scientific Q Exactive hybrid quadrupole Orbitrap mass spectrometer (ThermoFisher Scientific). The MS was operated in data‐dependent mode, automatically switching between MS and MS2 acquisition, with a mass resolution of 70 000 and 17 500, respectively. Mascot search engine was used for protein identification. MS raw files were searched against a database of 20 386 *Homo sapiens* sequences from UniProt. Protein scores (Table S5, Supporting Information) were derived from ion scores where individual ion scores > 15 indicated identity or extensive homology (p < 0.05). The protein lists identified in each group were overlapped with genes highly expressed in human skin fibroblasts (Table S5, Supporting Information).

### RNA‐Binding Protein Immunoprecipitation (RIP)

To validate the interaction between human *CircGLIS3(2)* and PCOLCE, an RIP assay was performed using Magna RIP RNA‐Binding Protein Immunoprecipitation Kit (Millipore, Burlington, MA). Human skin fibroblasts were lysed in RIP lysis buffer, and then 100 µl of whole cell extract was incubated with anti‐human PCOLCE antibody (sc‐73002, Santa Cruz Biotechnology) coated on A + G magnetic beads (Millipore) in RIP buffer. Normal rabbit IgG (Millipore) was used as a negative control. The samples were then incubated with proteinase K to digest protein, and the immunoprecipitated RNA was isolated. CircGLIS3 and *GAPDH* mRNA levels were detected by qRT‐PCR.

### Immunoprecipitation (IP)

Immunoprecipitation (IP) of CircGLIS3(2) protein and its protein interactome was done with the ANTI‐FLAG® M2 Affinity Gel. Briefly, cells were transfected with p‐CircGLIS3_FLAG and p‐CircGLIS3 plasmids. Crosslink reaction of the cells was done 48 h post‐transfection, cells were then treated with lysis buffer and incubated with ANTI‐FLAG® M2 Affinity Gel with its suggested protocol. Proteins were then eluted and sent for mass spectrometric analysis as described above (Table S7, Supporting Information).

### Single‐Cell RNA‐Sequencing Analysis of Fibroblasts in Human Skin Wounds

We conducted single‐cell RNA sequencing (scRNA‐seq) using the 10x Genomics platform on a series of acute wound samples from three human donors. Specimens were obtained from intact skin and the concentric wound edge across three distinct phases of wound healing: immediate inflammation (Day 1), proliferation (Day 7), and remodeling (Day 30). For fibroblast clusters, the average gene expression per cell was quantified, and genes were subsequently ranked based on their expression levels. Genes highly expressed in fibroblast were defined as mean_expression >2 (Table S6, Supporting Information).^[^ ^27^ ^]^

### Human *Ex Vivo* Wound Model

To evaluate the effect of *CircGLIS3(2)* in a physiologically relevant model of human skin wound healing, we employed a human *ex vivo* wound model.^[^ ^69^ ^]^ Human skin was obtained from abdominal reduction surgeries or thighplasty (donors 28–33 in Table S2, Supporting Information). The wounds were made using a 2 mm biopsy punch on the epidermal side of the skin (2‐4 wounds per donor), excised using a 6 mm biopsy punch, and subsequently transferred to a cell culture plate containing DMEM supplemented with 10% FBS and antibiotics (penicillin 50 U/l and streptomycin 50 mg ml^−1^; ThermoFisher Scientific). MaxSuppressor In Vivo RNA‐LANCEr II (Bioo Scientific, Austin, TX) or in vivo‐jetPEI® (Polyplusm, France) were used as transfection reagents. On the one hand, to knockdown CircGLIS3, the transfection reagents were mixed with 0.1 nmol siRNA targeting CircGLIS3 or a scrambled siRNA (Eurofins Genomics) in a volume of 5 µl per wound. On the other hand, to overexpress CircGLIS3, 3 µg of p‐CircGLIS3, p‐CircGLIS3_RNA, p‐CircGLIS3_protein or p‐Ctrl were added to the transfection reagent. The siRNA/plasmid‐lipid complexes were mixed 1:2 (volumes) in 30% pluronic F‐127 gel (Sigma–Aldrich). 15 µl mixture was topically applied on the wounds immediately after injury and 2 days later. Wound samples were collected six days after injury for gene expression and histological analysis.

### Murine In Vivo Wound Model

To investigate *CircGlis3* expression in murine wounds, skin and wound biopsies were collected from 15 C57BL/6 mice. Mice were housed individually for one week before and during the experiment. General anesthesia was performed using 3% isoflurane, and the back hair was shaved and treated with depilatory cream. On the same day, two 4‐mm full‐thickness wounds were created using a biopsy punch, with the excised skin saved as control. Mice were sacrificed before tissue collection. Wound‐edge tissue was collected with a 6‐mm biopsy punch on days 3 (n = 5), 7 (n = 5), and 10 (n = 5) post‐wounding. Mice received subcutaneous buprenorphine (30 µg kg^−1^) twice daily for the first two days post injury to relieve potential distress. Biopsies were used for RNA extraction and qRT‐PCR analysis. To assess the impact of *CircGlis3* on wound healing, either 0.1 nmol si‐CircGlis3 (n = 6) or si‐Control (n = 6) was topically applied to each wound (5 µL per wound) on Day 0 and Day 2 post‐injury. Wounds were covered with Tegaderm film. Wound images were captured on Days 0, 2, 4, and 6 to monitor healing. On Day 6, mice were euthanized, and wound tissues were harvested using a 6‐mm biopsy punch for RNA extraction, qRT‐PCR analysis, and histological examination via H&E staining. The experiment was approved by the North Stockholm Ethical Committee for Care and Use of Laboratory Animals (Stockholm, Sweden).

### Statistics

Data analysis was performed using R and GraphPad 8.4.0 (GraphPad Software). All quantitative data were presented as means ± SD. Normality and distribution of data were checked with the Shapiro‐Wilk test (p < 0.05 indicated data that did not pass the normality test). Comparison between two groups was performed with a two‐tailed Student's t‐test (parametric) or Wilcoxon test (non‐parametric, paired) or paired t test (paired). Comparison between more than two groups that contained paired data (matched samples or repeated measures) was made with RM one‐way ANOVA and Tukey's multiple comparisons test (parametric data) or Friedman test and Dunn's multiple comparisons test (non‐parametric data). Comparison between more than two groups with unpaired data was performed with Ordinary one‐way ANOVA and Dunnett's multiple comparisons test (parametric data) or Kruskal‐Wallis and Dunn's multiple comparisons test (non‐parametric data). p‐value < 0.05 was considered statistically significant.

The circRNA expression data of human skin and wound samples have been published and deposited in the Gene Expression Omnibus (GEO) database (GSE174661) and the analyzed dataset is presented at https://www.xulandenlab.com/humanwounds‐circrna.^[^ ^70^ ^]^ The microarray data of human dermal fibroblasts with *circGLIS3(2)* overexpression or knockdown can be accessed via GSE196260 at the GEO database. Source data are provided with this paper.

## Conflict of Interest

The authors declare no conflict of interest.

## Author Contributions

G.N. and M.A.T. contributed equally to this work. N.X.L., G.N., M.A.T., and P.S. performed conceptualization; G.N., M.A.T., Z.L., M.V., M.P., A.W., D.L., V.V., S.J.E., and P.S. performed methodology; G.N., M.A.T., J.G., X.B., Y.C., L.L., Q.W., D.L., Y.X., M.V., M.P., Z.L., S.O., L.Z., D.S., and A.V. performed investigation; G.N., M.A.T., and N.X.L. performed visualization; N.X.L. performed funding acquisition; N.X.L. performed project administration; N.X.L. and P.S. performed supervision; G.N., M.A.T., and N.X.L. wrote the original draft; G.N., M.A.T., J.G., X.B., Y.C., L.L., Q.W., D.L., Y.X., M.V., M.P., Z.L., S.O., L.Z., D.S., A.V, A.W., V.V., S.J.E., P.S., and N.X.L. wrote, reviewed and edited.

## Supporting information



Supporting Information

Characteristics of tissue donors

Information of donor skin samples

Differentially expressed circRNAs in human day‐7 wounds compared to skin

List of DEGs and signaling pathways in fibroblasts with abnormal circGLIS3 expression

Mass spectrometry analysis of protein interactome of CircGLIS3 RNA

Gene expression in human skin fibroblasts

Mass spectrometry analysis of protein interactome of CircGLIS3 protein

List of reagents used in this study_updated

## Data Availability

The data that support the findings of this study are available from the corresponding author upon reasonable request.
