# Supplementary material for: Collaborative Duality of CircGLIS3(2) RNA and Protein in human Wound Repair
Source: Adv Sci (Weinh). 2025 Apr 25;12(25):2416784. doi: 10.1002/advs.202416784 (PMC12224929; doi:10.1002/advs.202416784)
Supplement: Supplementary file 1 — Supporting Information [file ADVS-12-2416784-s003.docx]

Supplementary Materials for

Collaborative duality of CircGLIS3(2) RNA and protein in human wound repair

Guanglin Niu^1#^, Maria A. Toma^1#^, Jennifer Geara^1^, Xiaowei Bian^1^, Yongjian Chen^1^, Lihua Luo^1^, Qizhang Wang^1,2,3^, Yunting Xiao^4^, Manika Vij^1,5^, Minna Piipponen^1^, Zhuang Liu^1^, Sho Oasa^6^, Letian Zhang^1^, Dörte Schlesinger^7^, Ákos Végvári^8^, Dongqing Li^4^, Aoxue Wang^9^, Vladana Vukojević^6^, Simon J Elsässer^7^,Pehr Sommar^10^*, and Ning Xu Landén^1^*

*Correspondence to Ning Xu Landén, [ning.xu@ki.se](mailto:ning.xu@ki.se), and Pehr Sommar Pehr.sommar@regionstockholm.se

**Supplementary Materials**

Materials and Methods

Figure S1-S8

Table S1. Characteristics of tissue donors.

Table S2. Information of donor skin samples.

Table S3. Differentially expressed circRNAs in human day-7 wounds compared to the skin.

Table S4. List of differentially expressed genes and related signaling pathways in human fibroblasts with abnormal *CircGLIS3(2)* expression.

Table S5. Mass spectrometry analysis of protein interactome of *CircGLIS3(2)* RNA.

Table S6. Gene expression in human skin fibroblasts.

Table S7. Mass spectrometry analysis of protein interactome of CircGLIS3 protein.

Table S8. List of reagents used in this study.

**MATERIALS AND METHODS**

**Analysis of RNA-seq data and circRNA identification**

CircRNA identification and differential expression (DE) analysis of RNA-seq data was performed as previously reported (*1*) and presented as a browsable web resource (<https://www.xulandenlab.com/humanwounds-circrna>). Briefly, raw reads were first filtered using the Trimmomatic v0.36 package (*2*) to remove adaptor sequences and low-quality bases. Clean reads were then mapped to the human reference genome (GRCh38.p12) with the GENCODE genes annotation (version 31) using STAR v2.7.1 (*3*). The unmapped chimeric reads were used for circRNA identification using DCC software (*4*). CircRNAs were filtered by at least two back-spliced junction reads in a minimum of two samples. The expression of circRNAs was normalized to FPM (fragments mapped to back-splicing junctions per million mapped fragments) as previously described (*5*). CircRNAs were annotated based on chromosomal location and the overlap with three circRNA databases: circAtlas v2 (*6*), circBase (*7*), and CIRCpedia v2 (*8*). Differential circRNA analysis was performed by using the DESeq2 package (*9*). P-values calculated from the Wald test were adjusted by performing Benjamini-Hochberg (BH) multiple testing to estimate the false discovery rate (FDR). The differentially expressed circRNAs were defined as fold change greater than 2 and P value < 0.05. The most abundant DE circRNAs were selected based on mean normalized read count values higher than 1 (**Fig. 1B, Table S3**).

**RNA extraction and qRT-PCR**

Full-thickness skin and wound biopsies were homogenized using the TissueLyser LT (Qiagen, Hilden, Germany) prior to RNA extraction. Total RNA was isolated from human tissue, microdissected tissue, and isolated cells with the miRNeasy Mini Kit (Qiagen), and from cultured cells using TRIzol reagent (ThermoFisher Scientific). Gene expression analysis was performed using either human or mouse TaqMan expression assays (ThermoFisher Scientific) for *ACTA2*, *COL1A1*, *COL4A1*, and *FN1*, or SybrGreen expression assays (ThermoFisher Scientific) for *GLIS3* and *CircGLIS3(2)*. Expression levels were normalized to the housekeeping genes *18S*, *B2M*, *ACTB*, or *GAPDH*. Details of all primers/probes used in this study are provided in Table S8.

**Cell fractionation**

The cytoplasm and nucleus of dermal fibroblasts were separated using the Nuclear Extract Kit (Active Motif) according to the manufacturer’s instructions. Cells were washed with ice-cold PBS supplemented with phosphatase inhibitors and centrifuged at 200 × g for 5 minutes at 4°C to pellet the cells. For cytoplasmic fraction isolation, the cell pellet was resuspended in 500 µL of 1X Hypotonic Buffer and incubated on ice for 15 minutes to promote cell swelling. Subsequently, 25 µL of Detergent was added, and the mixture was vortexed. Cell lysis was confirmed under a microscope. The suspension was then centrifuged at 14,000 × g for 30 seconds at 4°C, and the supernatant, containing the cytoplasmic fraction, was collected. The remaining pellet was used to isolate the nuclear fraction. It was resuspended in 50 µL of Complete Lysis Buffer and incubated on ice for 30 minutes with gentle agitation at 150 rpm on a rocking platform. After a brief vortex, the suspension was centrifuged at 14,000 × g for 10 minutes at 4°C, and the supernatant, containing the nuclear fraction, was collected.

Mitochondria were isolated using the Mitochondria Isolation Kit for Cultured Cells (ThermoFisher Scientific). Cells were harvested by centrifugation at 850 × g for 2 minutes. The resulting pellet was resuspended in 800 µL of Mitochondria Isolation Reagent A, supplemented with protease inhibitors, and vortexed before being incubated on ice for 2 minutes. Following this, 10 µL of Mitochondria Isolation Reagent B was added, and the suspension was kept on ice for 5 minutes, with vortexing at 1-minute intervals. Next, 800 µL of Mitochondria Isolation Reagent C, also supplemented with protease inhibitors, was added, and the suspension was centrifuged at 700 × g for 10 minutes at 4°C. The supernatant was transferred to a new tube and further centrifuged at 12,000 × g for 15 minutes at 4°C. The resulting supernatant, representing the cytosolic fraction, was collected, while the pellet, containing the mitochondrial fraction, was washed with 500 µL of Mitochondria Isolation Reagent C supplemented with protease inhibitors and centrifuged at 12,000 × g for 5 minutes to obtain the purified mitochondrial fraction.

RNA was extracted from these fractions using TRIzol reagent (ThermoFisher Scientific). qRT-PCR was then performed to analyze the expression of CircGLIS3, GAPDH, MALAT1, and 16S rRNA.

**PCR and Sanger sequencing**

To confirm the circularity of both human and mouse *CircGLIS3*, PCR was performed using outward-facing primers to detect head-to-tail splice sites of *CircGLIS3*. Additionally, fibroblast RNA was digested with 1 unit RNaseR (Lucigen, Biosearch Technologies, Middleton, WI) per 1 μg RNA to enrich for circRNAs. Fifty nanograms cDNA from human or mouse fibroblasts (3T3 cells) were amplified with the divergent primers (listed in **Table S8**) using PCR Master Mix (2X) (ThermoFisher Scientific). The PCR products were then analyzed by DNA electrophoresis using E-Gel™ Agarose Gels with SYBR™ Safe DNA Gel Stain, 2% (Invitrogen, Waltham, MA, USA). The bands with expected sizes were cut out, and the DNA was purified using QIAquick Gel Extraction Kit (Qiagen). The purified DNA was then analyzed by Sanger sequencing using ABI 3730 PRISM^®^ DNA Analyzer at the KIgene core facility at Karolinska Institutet (Stockholm, Sweden).

**Gene expression microarray and analysis**

Expression profiling of human fibroblasts transfected with either scrambled siRNAs or plasmids for 24 hours was conducted using the human Clariom™ S assay (ThermoFisher Scientific) at the Bioinformatics and Expression Analysis (BEA) core facility at Karolinska Institutet. Briefly, total RNA was extracted using TRIzol, and RNA quality and quantity were assessed with the Agilent 2200 TapeStation using RNA ScreenTape and Nanodrop 1000. A total of 150 nanograms of RNA was used to prepare cDNA according to the GeneChip WT PLUS Reagent Kit labeling protocol. Standardized array processing procedures, including hybridization, fluidics processing, and scanning, were performed as recommended by Affymetrix.

Expression data were analyzed by using Transcriptome Analysis Console 4.0. Genes with a p-value less than 0.05 were considered as differentially expressed. KEGG pathway analysis was performed using Enrichr software (<https://maayanlab.cloud/Enrichr/>) to identify the top enriched biological pathways. Gene set enrichment analysis (GSEA) was performed to evaluate the enrichment of TGF- β1 target genes obtained from GSE79621 (*10*), **Table S4** among the genes regulated by *CircGLIS3* in fibroblasts, utilizing public software from the Broad Institute. The data have been deposited in the Gene Expression Omnibus under accession number GSE196260.

**Western blot**

Fibroblast protein lysates were extracted using radioimmunoprecipitation assay (RIPA) buffer (ThermoFisher Scientific) and analyzed by Western blotting. Antibody dilution ratios are provided in Table S8.

**Immunofluorescent staining**

Formalin-fixed paraffin-embedded tissue samples of human ex vivo wounds were deparaffinized and subjected to heat-induced antigen retrieval in Tris-EDTA buffer (pH = 9) at 98°C for 25 minutes. The tissue sections were then blocked with 5% bovine serum albumin (BSA; ThermoFisher Scientific) and incubated with the primary antibody overnight at 4°C. After washing with TBST (TBS containing 0.1% Triton-X), the sections were incubated with the secondary antibody for one hour. Following additional TBST washes, the sections were mounted using ProLong™ Diamond Antifade Mountant with DAPI (ThermoFisher Scientific).

Human dermal fibroblasts were fixed in 4% paraformaldehyde in PBS, blocked with 2.5% BSA in PBS, and permeabilized with 0.1% Triton-X in PBS. Cells were stained with primary antibodies overnight at 4°C, followed by incubation with the secondary antibody for one hour. The cells were mounted with ProLong™ Diamond Antifade Mountant with DAPI (ThermoFisher Scientific). Immunofluorescence-stained cells and tissue sections were visualized using a Nikon Eclipse Ni-E fluorescence microscope (Nikon) at 20X magnification.

**Confocal laser scanning microscopy imaging and quantification of CircGLIS3(2) protein nuclear localization**

Confocal laser scanning microscopy (CLSM) imaging was performed using the instrument LSM880 (Carl Zeiss) equipped with a 405 nm laser, 488 nm Ar-ion laser, and a water immersion objective lens (C-Apochromat, 40×, 1.2 N.A., Corr, Carl Zeiss), a gallium arsenide phosphide (GaAsP) detector and photomultiplier tube (PMT) detector. eGFP/CircGLIS3(2) immunostaining with the Donkey anti-Mouse IgG (H+L) Highly Cross-Adsorbed Secondary Antibody conjugated with Alexa Fluor 488 (Invitrogen, A-21202) and Hoechst33342/DAPI were excited using the 488 nm laser and 405 nm laser, respectively. The pinhole size was adjusted to 1AU (40 µm for eGFP/CircGLIS3(2) and 32 µm for Hoechst33342/DAPI). The fluorescence of eGFP/CircGLIS3(2) and Hoechst33342/DAPI were recorded by GaAsP detector (493 - 630 nm) and PMT detector (410 - 501 nm), respectively. To avoid the cross-talk artifact signal, a multi-track model was used. The imaging data was analyzed using ZEN software (Carl Zeiss). To quantify the nuclear localization of CircGLIS3, the average fluorescence intensity of eGFP and CircGLIS3(2) was calculated at the region of interests (ROIs) in the nucleus, *I*_nuc_ and the cytoplasm, *I*_cyto_. The ratio of fluorescence intensity nucleus to cytoplasm (N/C ratio) was calculated as follows:

$N/C ratio=\frac{I_{\text{nuc}}}{I_{\text{cyto}}}$ (1)

**Laser capture microdissection (LCM)**

Frozen tissue samples were sectioned at 10 μm thickness using a Microm HM355S rotary microtome (ThermoFisher Scientific, Carlsbad, CA) and stained with Mayer's hematoxylin (HistoLab, Stockholm, Sweden). Laser capture microdissection was performed using the Leica LMD7000 system (Leica Microsystems, Wetzlar, Germany).

**Magnetic-activated cell sorting (MACS)**

Fresh tissue samples were washed 2–3 times in PBS and incubated overnight at 4°C in 5 U/ml dispase (ThermoFisher Scientific) supplemented with antibiotics (50 U/ml penicillin and 50 mg/ml streptomycin; ThermoFisher Scientific). The epidermis was then separated from the dermis as previously described (*11*). The epidermis was cut into small pieces using scissors and digested in Trypsin/EDTA Solution (ThermoFisher Scientific) for 15 minutes at 37°C. CD45^-^ cells, primarily composed of keratinocytes, were then separated using CD45 Microbeads and MACS MS magnetic columns (Miltenyi Biotec, North Rhine-Westphalia, Germany). The dermis was incubated for 3 hours in the enzyme mix from the whole skin dissociation kit (Miltenyi Biotec) following the manufacturer's instructions and further processed using a Medicon tissue disruptor (BD Biosciences, Stockholm, Sweden). The resulting dermal cell suspension was incubated with CD90 Microbeads, and CD90^+^ fibroblasts were isolated using MACS MS magnetic columns according to the manufacturer's instructions (Miltenyi Biotec).

***In situ* hybridization**

A *CircGLIS3(2)* probe targeting the *CircGLIS3(2)* BSJ, a negative control probe targeting the Bacillus subtilis dihydrodipicolinate reductase (DapB) gene, and a positive control probe targeting Homo sapiens peptidylprolyl isomerase B (cyclophilin B; PPIB) mRNA were designed and synthesized by Advanced Cell Diagnostics (ACD, Newark, CA). Human fibroblasts were cultured on slides and fixed in cold 4% formaldehyde for 15 minutes. After dehydration with 50%, 70%, and 100% ethanol, the cells were incubated with Protease III (ACD) at room temperature for 20 minutes. The slides were then hybridized with either the *CircGLIS3(2)* probe or the negative and positive control probes for two hours at 40°C using the HybEZ™ II Hybridization System with the BaseScope™ Reagent Kit v2 – RED Assay (ACD). The hybridization signals were amplified through sequential hybridization of amplifiers and visualized using chromogenic staining with Fast RED dye. Cells were counterstained with 50% hematoxylin for 2 minutes. Visualization was performed using brightfield microscopy on a Nikon Eclipse Ni-E microscope (Nikon, Amstelveen, Netherlands) at 20X and 40X magnification.

**Combination of fluorescent *in situ* hybridization and immunofluorescence analysis**

Fluorescent *in situ* hybridization (FISH) was performed as previously described (*12*). Briefly, a probe targeting the junction site of *CircGLIS3(2)* (**Table S8**) was designed and synthesized as a single-stranded DNA oligonucleotide conjugated with digoxigenin (Integrated DNA Technologies). Cells were fixed in 4% paraformaldehyde, permeabilized with 0.3% Triton-X in PBS, and blocked with PBS containing 0.1% Tween and 10% BSA. Hybridization was carried out overnight at 42°C. For detection, samples were incubated with an anti-digoxigenin-HRP antibody, and the signal was amplified using the Alexa Fluor 647 Tyramide Reagent (Life Technologies). Immunofluorescence was then performed to determine the cellular localization of PCOLCE protein using an anti-PCOLCE antibody (Santa Cruz, catalog no. 10D9: sc-73002). Samples were mounted with SlowFade Diamond Antifade Mountant containing DAPI (Life Technologies).

**Cell culture and functional studies**

Human primary dermal fibroblasts, adult (HDFa; Cascade Biologics, Portland, OR) were cultured in Medium 106 (Cascade Biologics) supplemented with 10% Low Serum Growth Supplement (LSGS) and 1% penicillin/ streptomycin at 37°C in 5% CO2 (ThermoFisher Scientific).

Dermal fibroblasts were isolated from adult human skin from abdominal or thigh reduction plastic surgery (n = 2) (donors 31 and 32 in **Table S2**). Six-mm full-depth skin biopsies were collected with a punch knife and washed with PBS. The tissues were then placed in a culture plate and left to attach to the bottom. Dulbecco’s Modified Eagle Medium (DMEM) supplemented with 10% heat-inactivated fetal bovine serum (FBS) and 1% penicillin-streptomycin (ThermoFisher Scientific) was then added to the culture plate, which was kept at 37 °C in 5% CO_2_. Fibroblasts grew out from the tissues, and the culture became confluent in approximately two weeks. Cells were passaged once for expansion. Passage two fibroblasts were cryopreserved. Fibroblasts in passages three and four were used in this study.

NIH/3T3 mouse embryonic fibroblasts (CRL-1658™; ATCC, Manassas, VA) were cultured in DMEM medium supplemented with 10% heat-inactivated FBS and 1% penicillin-streptomycin at 37°C in 5% CO2 (ThermoFisher Scientific). HEK293T cells were cultured in DMEM medium supplemented with 10% FCS and 1% penicillin-streptomycin at 37°C in 5% CO2.

To evaluate RNA stability, we incubated human fibroblasts with Actinomycin-D 5 μg/ml for up to 24 h. To study the mechanism regulating CircGLIS3 expression, we treated human fibroblasts with IL-1α (20 ng/ml), IL-6 (50 ng/ml), IL-8 (50 ng/ml), IL-22 (30 ng/ml), IL-36α (100 ng/ml), TNF-α (50 ng/ml), TGF-β1 (20 ng/ml), TGF-β2 (10 ng/ml), TGF-β3 (20 ng/ml), BMP-2 (100 ng/ml), EGF (20 ng/ml), IGF-1 (20 ng/ml), FGF-2 (30 ng/ml), VEGFA (20 ng/ml), HB-EGF (20 ng/ml) or PBS as control for 24 h and CircGLIS3 expression was analyzed by qRT-PCR. All these cytokines and growth factors were purchased from either ImmunoTools (Frieshoyte, Germany) or R&D Systems (Minneapolis, MN) (**Table S8**).

To study the functions of *CircGLIS3(2)* in fibroblasts, we performed knockdown and overexpression experiments. For knockdown, cells at 60–70% confluence were transfected with 20 nM of siRNA targeting *CircGLIS3(2)* or a scrambled siRNA for 24 or 48 hours using Lipofectamine™ 3000 (ThermoFisher Scientific). For circRNA-protein binding site blockage, cells at 60–70% confluence were transfected with 20 nM of antisense oligos (ASOs) targeting *CircGLIS3(2)* or a scrambled antisense oligo (ASO) for 24 hours using Lipofectamine™ RNAiMAX Reagent (ThermoFisher Scientific). To overexpress *CircGLIS3(2)* (RNA, protein, or both), fibroblasts at 80–90% confluence were transfected with *CircGLIS3(2)* overexpression plasmids using Lipofectamine™ 3000 for 48 hours. To evaluate the effect of *CircGLIS3(2)* on TGF-β signaling, human fibroblasts with either *CircGLIS3(2)* depletion or overexpression, as well as 3T3 cells with *CircGlis3* knockdown, were stimulated with 5 ng/ml TGF-β1 (R&D Systems) for 24 hours following 24 hours of transfection. The corresponding amount of TGF-β1 reconstitution buffer was used as a vehicle negative control. These cells were then subjected to qRT-PCR analysis. To assess the turnover of PCOLCE and BTF3 proteins, human fibroblasts were treated with MG132, cycloheximide, Ubiquitin E1 inhibitor (E1), UbcH13 inhibitor (E2), Heclin (E3), and Bafilomycin A1 (L1), with concentrations listed in Table S8. To activate CircGLIS3 translation, we treated human fibroblasts with bortezomib, 4EGI-1, thapsigargin (TG), and tunicamycin (Table S8), as well as applying heat shock and hypoxia. To inhibit the IL-1α and TGF-β1 pathways, human fibroblasts were treated with p38, JNK, and ERK inhibitors, as well as Asiaticoside, at the concentrations listed in **Table S8**.

**Detection of nascent RNAs**

Fibroblast cells were seeded in 12 well plates. After 24 h, the cells were treated with IL-1α for 2h, 4h and 24 h. The nascent RNA synthesis assay was performed by using the Click-iT™ Nascent RNA Capture Kit (ThermoFisher Scientific) following the manufacturer’s protocol.

**Plasmid construction**

The *CircGLIS3(2)* overexpression plasmid was constructed with the help of Guangzhou Geneseed Biotech Co. (Guangzhou, China). In brief, the pLC5-ciR vector, which includes front and back circular frames for the circularization of the transcripts, was used as the backbone plasmid. The front circular frame contains an endogenous flanking genomic sequence with the EcoRI restriction site, and the back circular frame contains part of the inverted upstream sequence with the BamHI restriction site. The cDNA encoding *CircGLIS3(2)* in HEK293T cells was amplified using the primers listed in **Table S8**. The amplicon, which contained an EcoRI site, the *CircGLIS3(2)* linear sequence with the corresponding splice sites, and a BamHI site, was then cloned into the pLC5-ciR backbone vector between the two frames. Vector construction was verified by Sanger sequencing. A mock vector containing only a nonsense sequence between the two circular frames was used as a control plasmid.

The *CircGLIS3(2)* overexpression plasmids with or without MS2 hairpins were constructed with the help of Creative Biogene Biotechnology (Shirley, NY). In brief, the cDNA encoding *CircGLIS3(2)* or *CircGLIS3(2)-MS2* were subcloned into the pLO-circRNA backbone by restriction digestion with EcoRI and BamHI and ligation with T4 DNA ligase. CircGLIS3 overexpression vector (p-CircGLIS3) was constructed by subcloning the DNA sequence corresponding to *CircGLIS3(2)* and its endogenous flanking region, which also included complementary circular frames needed for circularization, into a plasmid expression cassette. A plasmid containing a 3×FLAG sequence before the predicted ORF stop codon (p-CircGLIS3_FLAG) and a plasmid containing a 3×FLAG sequence before the stop codon as well as a ATG mutation of the ORF (p-CircGLIS3_FLAG_mut) were constructed based on the modification of p-CircGLIS3. ATG (from the ORF) mutated plasmid was constructed based on p-CircGLIS3 which only overexpresses the *CircGLIS3(2)* RNA itself, not the protein (p-CircGLIS3_RNA). On the contrary, the ORF sequence was subcloned to the overexpression vector which only overexpresses the CircGLIS3(2) protein, not the RNA (p-CircGLIS3_protein). A Protein binding site (BTF3 and CircGLIS3(2) protein) mutated plasmid was constructed based on p-CircGLIS3 which overexpresses the CircGLIS3(2) protein with binding site mutation (p-CircGLIS3_FLAG_binding_mutant (**Fig. S2D**)).Dual luciferase plasmid was a gifted from Vincent Mauro (Addgene plasmid # 51733), we subclone the predicted IRES fragment between Firefly and Renilla region to make the IRES testing vector and multi cloning sites (MCS) as a control plasmid. Vector construction was verified by Sanger sequencing, and the primers used are listed in **Table S8**.

**Luciferase assay**

To evaluate the effect of *CircGLIS3(2)* on the responsiveness of human fibroblasts to TGF-β1 stimulation, we used a TGF-β reporter plasmid pSBE4-Luc (Addgene, plasmid #16495). This plasmid contains four tandem copies of the Smad binding sites, which drive the transcription of the Firefly luciferase reporter gene (*13*). pBV-Luc (Addgene, plasmid #16539), a luciferase reporter plasmid with very low basal activity, was used as a negative control (*14*). Human dermal fibroblasts were co-transfected with the luciferase reporters (200 ng/ml), together with 20 nM siRNA targeting *CircGLIS3(2)* or scrambled control, using the Lipofectamine™ 3000 (ThermoFisher Scientific). One day later, the transfected cells were treated with 5 ng/ml TGF-β1 (R&D Systems) for 24 h. To study IRES activity, vectors containing predicted IRES and control fragments were transfected into HEK293 cells using Lipofectamine™ 3000 (ThermoFisher Scientific) for 48 hours. Luciferase activity was analyzed using the Dual-Luciferase® Reporter Assay System and measured with the GloMax®-Multi Detection System (Promega, Madison, WI).

***In silico* analysis of interaction domains in the *CircGLIS3(2)* RNA–PCOLCE and CircGLIS3(2) Protein–BTF3 complexes**

AlphaFold3 (<https://alphafoldserver.com>)(*15*) was used to predict the three-dimensional structures of the *CircGLIS3(2)* RNA–PCOLCE and CircGLIS3(2) Protein–BTF3 complexes and highlight potential interaction interfaces. The amino acid sequences of BTF3 and PCOLCE were retrieved from UniProt (<https://www.uniprot.org/>) and submitted alongside *CircGLIS3(2)* sequence to AlphaFold3 for complex structure prediction. The resulting structures were analyzed using PyMOL (*16*). To identify atomic-level contacts and putative binding regions within *CircGLIS3(2)*, PyMOL’s "find any contacts" and "find any atoms" functions were applied. Additionally, for the CircGLIS3(2)-BTF3 protein-protein complex, interface residues were further analyzed using PRODIGY,which predicted interface residues, binding free energy (ΔG), and the dissociation constant (Kd) at 25°C(*17*).

**Cellular thermal shift assay (CETSA) and immunoblot**

Human dermal fibroblasts transfected with either 20 nM si-CircGLIS3 or scrambled siRNA (Eurofins Genomics, Ebersberg, Germany) for 48 hours and treated with 5 ng/ml TGF-β1 (R&D Systems) were rinsed and pelleted in PBS. Cells were resuspended in PBS supplemented with protease inhibitors (Roche, Basel, Switzerland) and counted to normalize equal cell density between conditions. Cells were lysed with three rounds of freeze-thaw cycles by incubating for three minutes on dry ice and three minutes in a water bath at 37°C, followed by centrifugation at 16,000 × *g* for 20 min at 4°C. The supernatant was then aliquoted into PCR tubes and heated individually at different temperatures (range: 55-90°C, 2.5°C increments) for 3 min in a gradient thermal cycler ProFlex PCR System (ThermoFisher Scientific) and immediately cooled down at room temperature. After centrifugation (20,000 × *g* for 20 min at 4°C), the supernatant was transferred to a new tube and prepared for immunodetection with an anti-human PCOLCE antibody (1:25; catalog sc-73002; Santa Cruz Biotechnology) with Protein Simple Jess/Wes capillary-based system (Bio-Techne, Minneapolis, MN) according to manufacturer instructions.

**Cell growth assay and cell cycle analysis**

Cell growth was measured by Real-time incucyte S3 (Sartorius AG, Germany) according to the instructions. Briefly, cells were seeded in 12-well plates (Sarstedt) at low density (typically 20.000 cells/well) and allowed to adhere overnight. Imaging was performed every two hours using the Incucyte ZOOM system. Cell proliferation was subsequently quantified using the IncuCyte ZOOM software (Sartorius). For cell cycle analysis, cells were first fixed with 4% PFA and then stained with DAPI (10µg/ml) and sorted by FACSVerse (BD biosciences). The cell cycle phases were analyzed by flowjo (BD biosciences).

**Supplementary figures:**

**
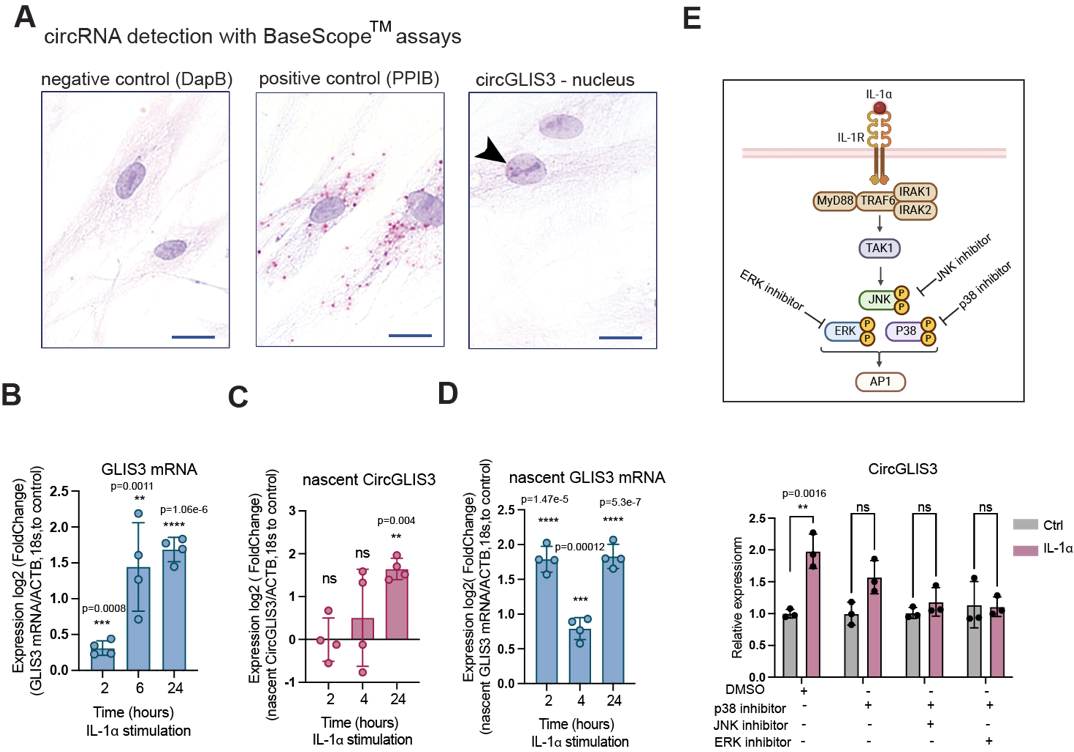
**

**Figure S1. Subcellular localization and expression regulation of *CircGLIS3(2)*. ** **A** RNA *in situ* hybridization using the BaseScope^TM^ assay on human dermal fibroblasts The images display the signals from the Fast RED dye for the negative control probe (detecting DapB), the positive control probe (detecting *PPIB* mRNA), and the probe targeting *CircGLIS3(2)* BSJ. The black arrow indicates the *CircGLIS3(2)* signal. Scale bar = 20 μm. qRT-PCR analysis was conducted to measure *GLIS3* mRNA (**B**), nascent *CircGLIS3(2)* (**C**) and nascent *GLIS3* mRNA (**D**) in fibroblasts treated with IL-1α (20 ng/μl) for 2-24 hours (n=4), (**E**) Schematics of inhibitors targeting the IL-1α pathway (up), qRT-PCR analysis of *CircGLIS3(2)* in fibroblasts co-treated with IL-1α and p38/JNK/ERK inhibitors (n=3) (down). Gene expression data were normalized to the housekeeping genes *ACTB* and *18S and* are presented relative to the levels in untreated cells. Data are presented as means ± SD. ns P ≥ 0.05, *P<0.05 **P<0.01, ***P<0.001, and ****P<0.0001 by Student’s t-test (**B**-**D**) or two-way ANOVA and Tukey's multiple comparisons test (**E**).


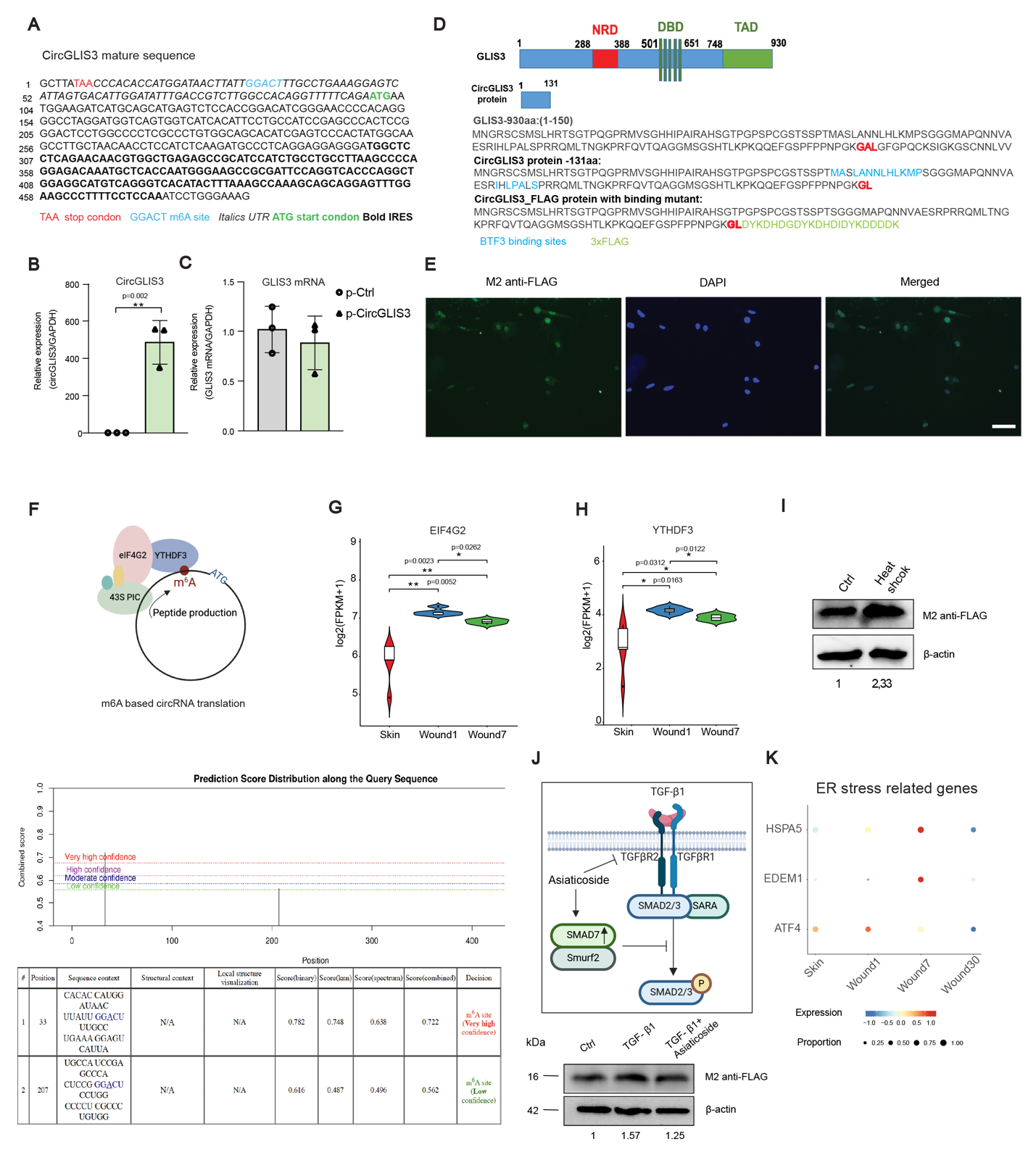


**Figure S2. Characterization of *CircGLIS3(2)* encoded protein. (A)** Thesequence of *CircGLIS3(2)*. qRT-PCR analysis of *CircGLIS3(2)* (**B**) and GLIS3 mRNA (**C**) in human skin fibroblasts transfected with p-CircGLIS3 or control vector (n=3). (**D**) Schematic representation of protein domain structure of *GLIS3,* *CircGLIS3(2)*-encoded and *CircGLIS3(2) FLAG* with BTF3binding sites mutant proteins. NRD: Negative Regulatory Domain; DBD: DNA binding Domain; TAD: Transactivation Domain. (**E**) Immunofluorescence staining of CircGLIS3-FLAG protein in fibroblasts transfected with p-CircGLIS3_FLAG. Scale bar=100 μm. (**F**) Prediction of m6A site in *CircGLIS3(2)* sequence byusing the SRAMP tool. RNA sequencing data showing the expression of EIF4G2 (**G**) and YTHDF3 (**H**) in skin and wound tissues (n=5). Western blot analysis of CircGLIS3-FLAG protein in HEK293 cells treated with heat shock (**I**); (**J**) Schematics of Asiaticoside targeting the TGF- pathway (up), western blot analysis of CircGLIS3-FLAG protein in HEK293 cells treated with TGF- and Asiaticoside (down). (**K**) Single-cell RNA sequencing data showing the expression of ER stress-related genes in fibroblasts during the wound healing process. Data are presented as means ± SD. ns P ≥ 0.05, *P<0.05 and **P<0.01 by Student’s t-test (**B**, **C**) or one-way ANOVA and Tukey's multiple comparisons test (**G**, **H**).

**
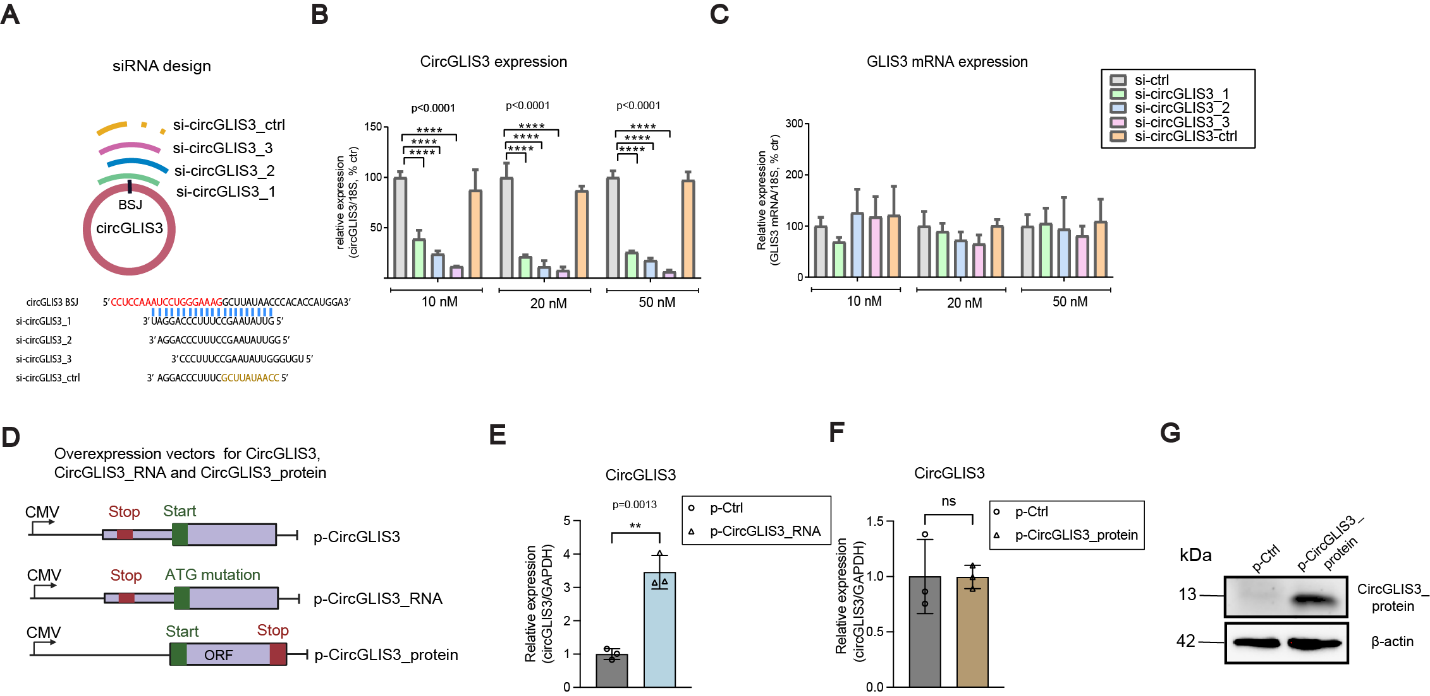
**

**Figure S3. Modulation of *CircGLIS3(2)* expression level in human dermal fibroblasts.** (**A**) Illustration of siRNA design for silencing *CircGLIS3(2)* expression. Three siRNAs were designed to target the back-splicing junction (BSJ) sequence of *CircGLIS3(2)*. The red and black sequences represent the corresponding 5' and 3' exon sequences that form the BSJ. A non-targeting siRNA (si-ctrl) and a siRNA that partially targets the BSJ (si-circGLIS3_ctrl with mismatched sequences shown in brown) were used as controls. qRT-PCR analysis of *CircGLIS3(2)* (**B**) and *GLIS3* mRNA (**C**) expression in human fibroblasts transfected with 10, 20, or 50 nM of siRNAs for 24 hours (n=3-4). (**D**) Schematic representation for p-CircGLIS3, p-CircGLIS3_RNA, and p-CircGLIS3_protein overexpression vectors. qRT-PCR analysis of *CircGLIS3(2)* expression in human fibroblasts transfected with p-CircGLIS3_RNA or p-Ctrl vectors (n=3) (**E**), and in fibroblasts transfected with p-CircGLIS3_Protein or p-Ctrl vectors for 48 hours (n=3) (**F**). (**G**) Western blot analysis of CircGLIS3- protein in HEK293 transfected with the p-CircGLIS3_protein vector. Data are presented as means ± SD. ns P ≥ 0.05, **P<0.01 and ****P<0.0001 by Two way ANOVA and Dunnett's multiple comparisons (**B**) or one-way ANOVA and Tukey's multiple comparisons test (**C**) or Student’s t-test (**E**, **F**).


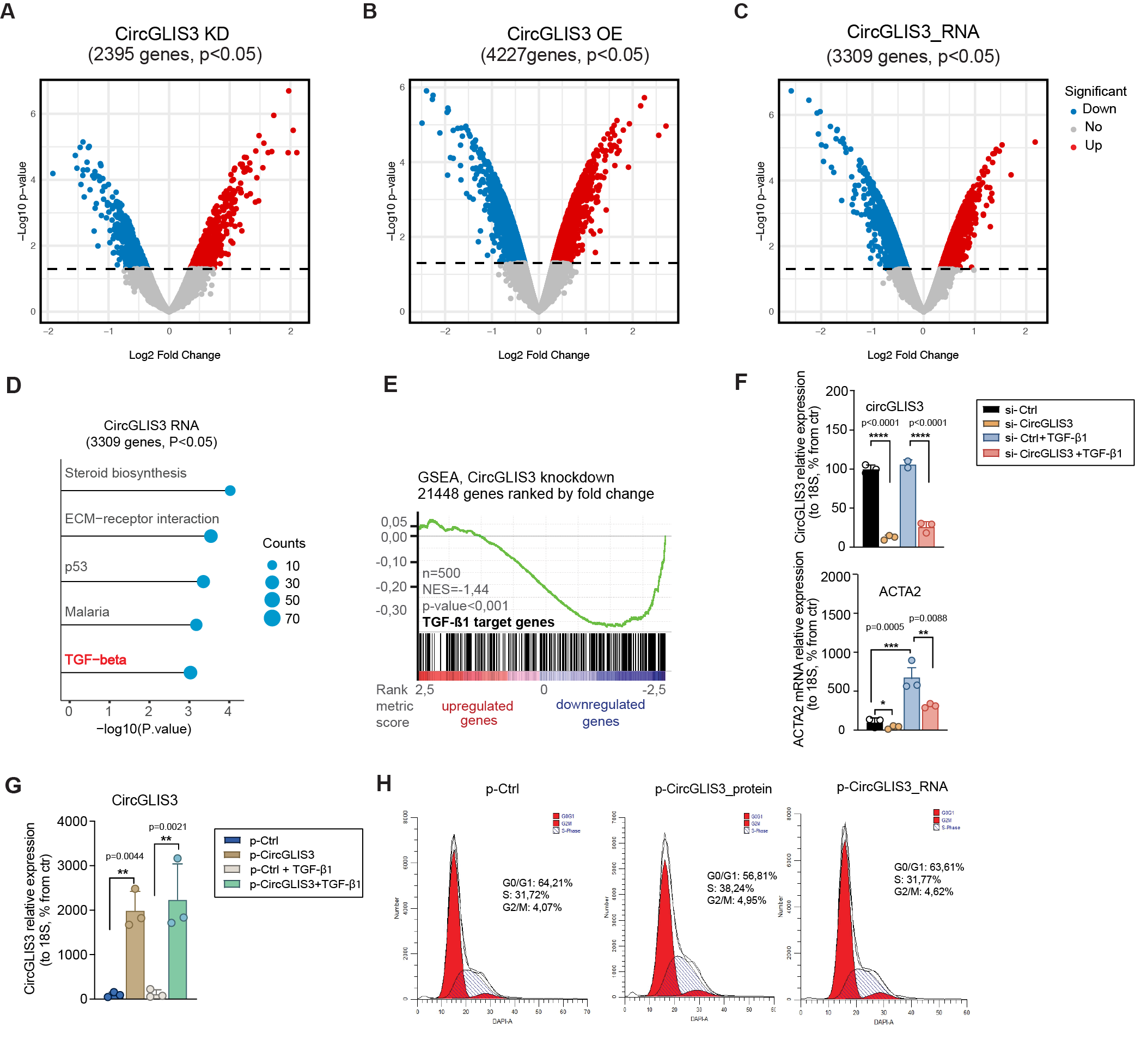


**Figure S4. Divergent functions of *CircGLIS3(2)* RNA and protein in wound fibroblasts.**  Volcano plots illustrate the differentially expressed genes (DEGs) with a p-value < 0.05 for *CircGLIS3(2)* knockdown (KD) (**A**), *CircGLIS3(2)* overexpression (OE) (**B**), and *CircGLIS3(2)* RNA OE (**C**) in human skin fibroblasts. (**D**) KEGG enrichment analysis was performed for the DEGs in fibroblasts with *CircGLIS3(2)* RNA OE. (**E**) Gene Set Enrichment Analysis (GSEA) evaluated the enrichment of TGF-β1 signaling-related genes in the microarray data of human fibroblasts with *CircGLIS3(2)* KD. (**F**) qRT-PCR analysis of *CircGLIS3(2)* and *ACTA2* mRNA expression in fibroblasts transfected with either si-ctrl or si-CircGLIS3 and treated with TGF-β1. (n=3). (**G**) qRT-PCR analysis of *CircGLIS3(2)* in fibroblasts with *CircGLIS3(2)* OE and TGF-β1 treatment. (**H**) Cell cycle analysis was performed on human dermal fibroblasts transfected with p-CircGLIS3_protein, p-CircGLIS3_RNA, or p-Ctrl vectors (n=3). Data are presented as means ± SD. ns P ≥ 0.05, *P<0.05 **P<0.01, ***P<0.001, and ****P<0.0001 by one-way ANOVA and Tukey's multiple comparisons test (**F**, **G**).

**
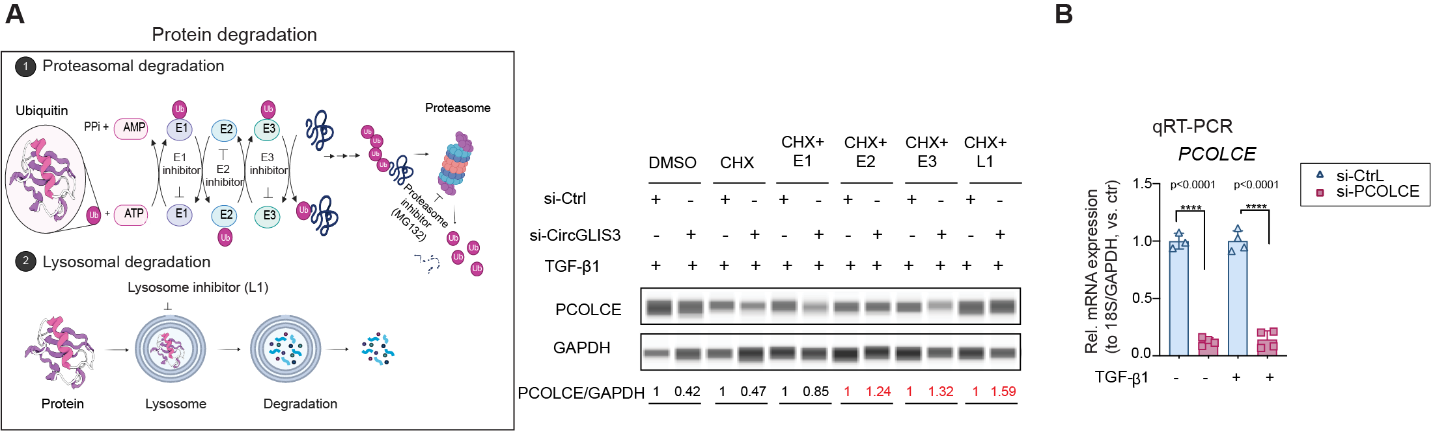
**

**Figure S5. *CircGLIS3(2)* RNA stabilizes PCOLCE protein.** (**A**) Illustration of protein degradation process. Simple Western of PCOLCE in fibroblasts with *CircGLIS3(2)* depletion and TGF-β1 treatment for 24 hours and then treated with cycloheximide (CHX) and inhibitors for proteasomal degradation (E1, E2, E3) or for lysosomal degradation (L1). The levels of PCOLCE relative to GAPDH were quantified. (**B**) qRT-PCR analysis of *PCOLCE* mRNA in fibroblasts transfected with either si-ctrl or si-PCOLCE and stimulated with TGF-β1 for 24 hours (n=3-4). Data are presented as means ± SD. ****P < 0.0001 by one-way ANOVA and Tukey’s multiple comparisons test.


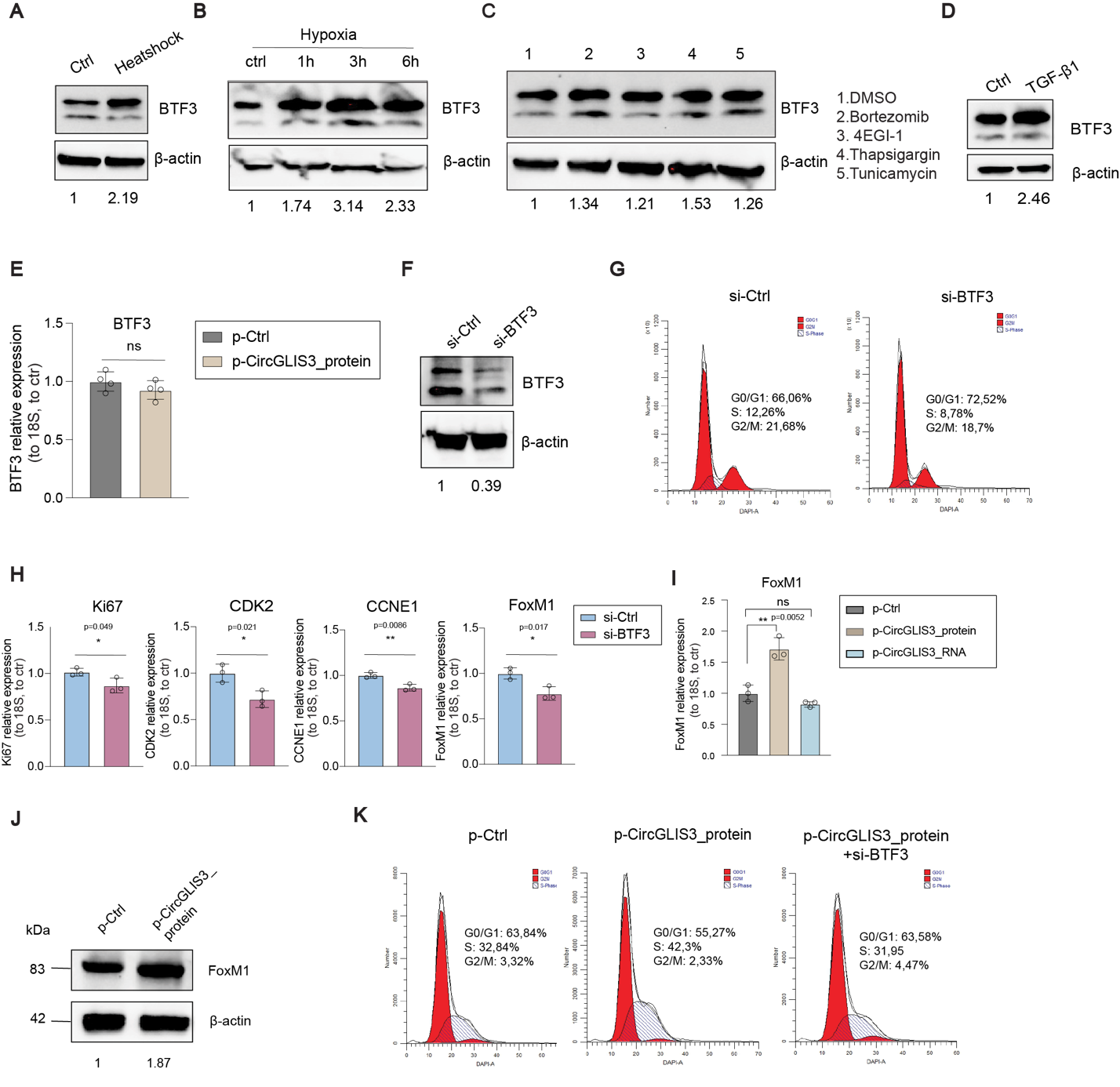


**Figure S6.** ***CircGLIS3(2)* encoded protein binds BTF3 in cell cycle regulation.** Western blot analysis of BTF3 protein in HEK293 cells under heat shock (**A**), hypoxia (**B**), treated with chemicals that induce ER stress (**C**) or TGF- (**D**). (**E**) qRT-PCR analysis of *BTF3* mRNA in fibroblasts transfected with p-CircGLIS3_protein and p-Ctrl vectors (n=4). (**F**) Western blot analysis of BTF3 protein in fibroblasts transfected with si-BTF3 or control siRNA. (**G**) Cell cycle analysis of human fibroblasts transfected with BTF3 siRNA. (**H**) qRT-PCR analysis of cell proliferation regulators in fibroblasts transfected with either si-ctrl or si-BTF3 (n=3). (**I**) qRT-PCR analysis of FoxM1 in human fibroblasts transfected with p-CircGLIS3_protein, p-CircGLIS3_RNA, or p-Ctrl vectors (n=3). (**J**) Western blot analysis of FoxM1 in fibroblasts transfected with p-CircGLIS3_protein or p-Ctrl. (**K**) Cell cycle analysis of human fibroblasts transfected with p-CircGLIS3_protein and BTF3 siRNA. Data are presented as means ± SD. ns P ≥ 0.05, *P<0.05 and **P<0.01 by Student’s t-test (**E**, **H**, **I**).


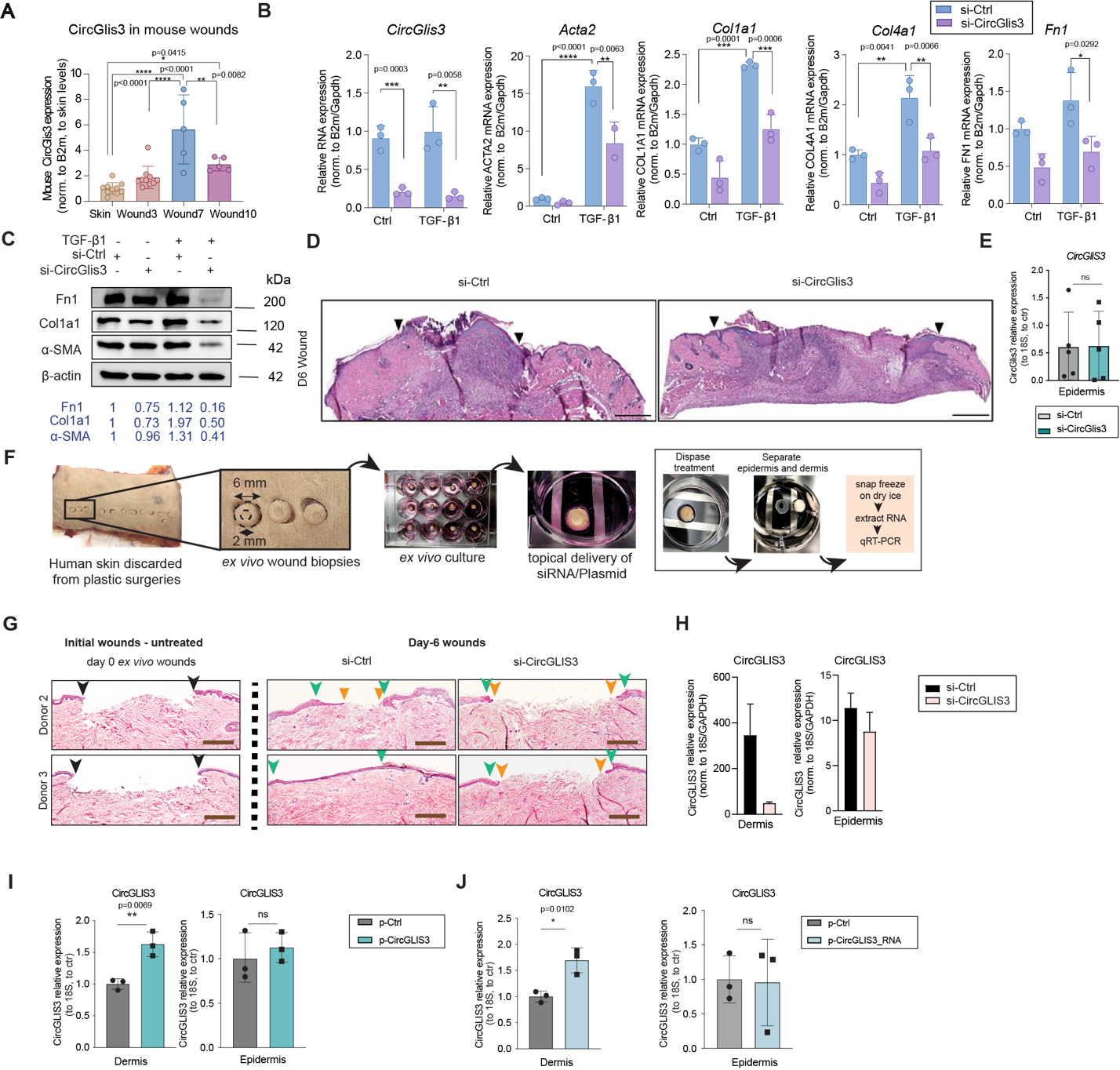


**Figure S7. *CircGLIS3(2)* is essential for wound repair.** (**A**) qRT-PCR analysis of *CircGlis3* expression in skin and days 3, 7, and 10 acute wounds of C57BL/6 mice (n=15). (**B**) qRT-PCR analysis of *CircGlis3, Acta2, Col1a1, Col4a1* and *Fn1* expression in murine fibroblasts (3T3) transfected with either mouse si-Ctrl or si-CircGlis3 and stimulated with TGF-β1 for 24 hours (n=3). (**C**) Western blot analysis of Fn1, Col1a1, α-SMA expression in murine fibroblasts (3T3) transfected with either mouse si-Ctrl or si-CircGlis3 and stimulated with TGF-β1 for 24 hours (**D**) Representative images of Hematoxylin and eosin (H&E) staining of Day-6 murine wounds treated with either si-Ctrl or si-CircGlis3. Arrowheads indicate the initial wound edge, and dashed lines indicate newly formed epidermis. Scale bar = 500 μm. (**E**) qRT-PCR analysis of epidermal *CircGlis3* expression in murine wounds. (**F**) Schematic overview of the workflow for the human ex vivo wound model. (**G**) H&E staining of day 0 and day 6 human *ex vivo* wounds. Black arrows denote the initial wound edges at day 0, green arrows indicate the wound edges at day 6, and orange arrows highlight the newly formed epidermis. Scale bar = 500 μm. qRT-PCR analysis of *CircGLIS3(2)* expression in the epidermis and dermis of human *ex vivo* wounds treated with si-CircGLIS3(2) (**H**), or p-CircGLIS3 (**I**), or p-CircGLIS3_RNA (**J**). Data are presented as means ± SD. ns P ≥ 0.05, *P<0.05, **P<0.01, ***P<0.001 and ****P<0.0001 by one way ANOVA and Tukey's multiple comparisons test (**A**), or two-way ANOVA and Multiple comparisons (**B**), or Student’s t-test (**E**, **I**, **J**).
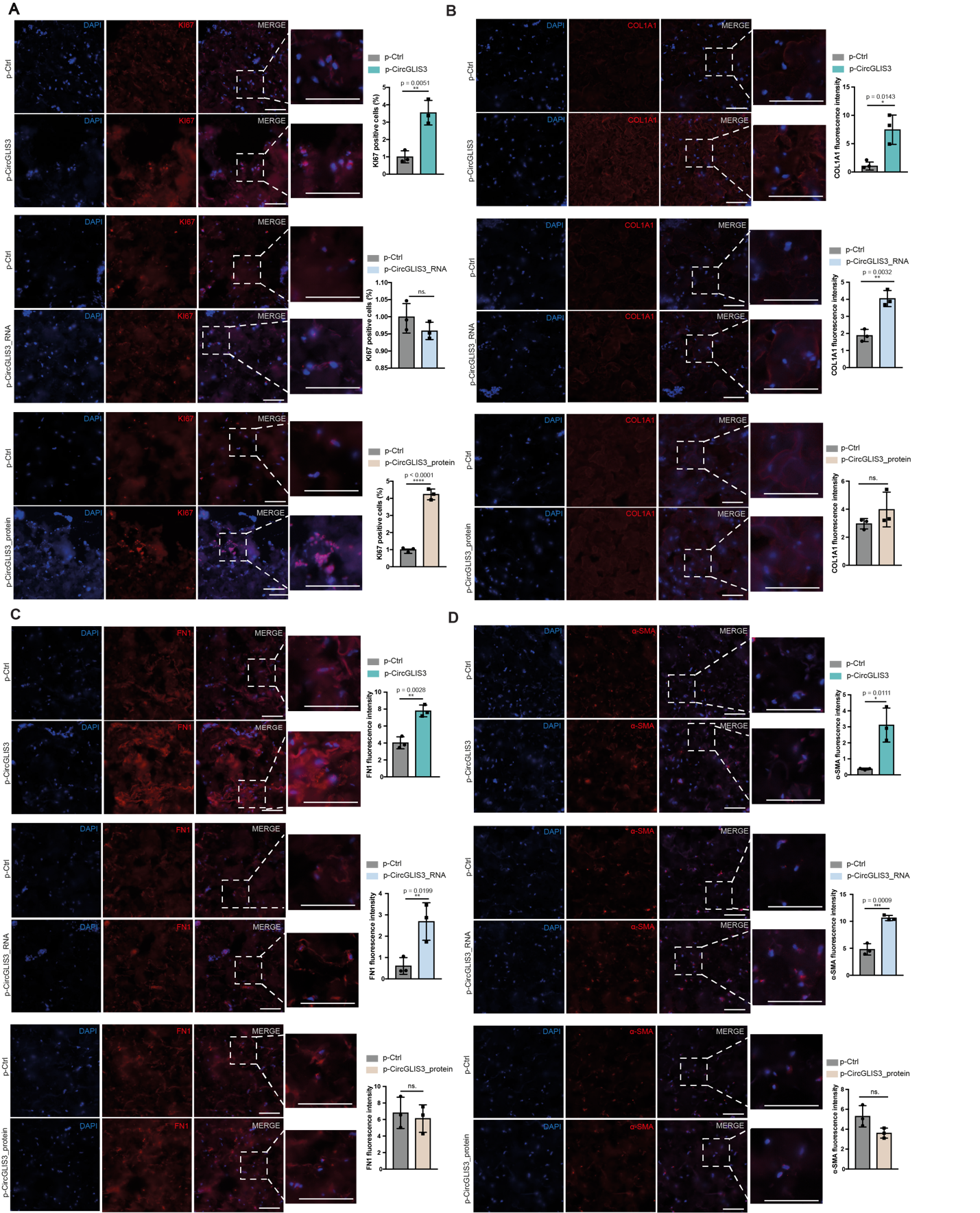


**Figure S8. Immunofluorescence (IF) analysis of human *ex vivo* wounds.** Human *ex vivo* wounds were treated with p-CircGLIS3, or p-CircGLIS3_RNA, or p-CircGLIS3_protein or control vectors(n=3 /group). Representative images of immunofluorescence analysis of Ki67 (**A**), COL1A1(**B**), FN1(**C**) and α-SMA(**D**). scale bar = 100 μm. The signal intensity was quantified. Data are presented as means ± SD. ns P ≥ 0.05, *P<0.05 **P<0.01, ***P<0.001, and ****P<0.0001 by Student’s t-test (**A**, **B**, **C**, **D**).

**Supplementary references:**

1. M. A. Toma *et al.*, Circular Rna Signatures Of Human Healing And Non-Healing Wounds. *J Invest Dermatol*, (2022).

2. A. M. Bolger, M. Lohse, B. Usadel, Trimmomatic: a flexible trimmer for Illumina sequence data. *Bioinformatics* **30**, 2114-2120 (2014).

3. A. Dobin *et al.*, STAR: ultrafast universal RNA-seq aligner. *Bioinformatics* **29**, 15-21 (2013).

4. Y. Liao, G. K. Smyth, W. Shi, featureCounts: an efficient general purpose program for assigning sequence reads to genomic features. *Bioinformatics* **30**, 923-930 (2014).

5. J. Cheng, F. Metge, C. Dieterich, Specific identification and quantification of circular RNAs from sequencing data. *Bioinformatics* **32**, 1094-1096 (2016).

6. W. Wu, P. Ji, F. Zhao, CircAtlas: an integrated resource of one million highly accurate circular RNAs from 1070 vertebrate transcriptomes. *Genome Biol* **21**, 101 (2020).

7. P. Glazar, P. Papavasileiou, N. Rajewsky, circBase: a database for circular RNAs. *RNA* **20**, 1666-1670 (2014).

8. R. Dong, X. K. Ma, G. W. Li, L. Yang, CIRCpedia v2: An Updated Database for Comprehensive Circular RNA Annotation and Expression Comparison. *Genomics Proteomics Bioinformatics* **16**, 226-233 (2018).

9. M. I. Love, W. Huber, S. Anders, Moderated estimation of fold change and dispersion for RNA-seq data with DESeq2. *Genome Biol* **15**, 550 (2014).

10. S. Bhattacharyya *et al.*, Tenascin-C drives persistence of organ fibrosis. *Nat Commun* **7**, 11703 (2016).

11. S. Cheuk *et al.*, CD49a Expression Defines Tissue-Resident CD8(+) T Cells Poised for Cytotoxic Function in Human Skin. *Immunity* **46**, 287-300 (2017).

12. I. F. Hall *et al.*, Circ_Lrp6, a Circular RNA Enriched in Vascular Smooth Muscle Cells, Acts as a Sponge Regulating miRNA-145 Function. *Circ Res* **124**, 498-510 (2019).

13. L. Zawel *et al.*, Human Smad3 and Smad4 are sequence-specific transcription activators. *Mol Cell* **1**, 611-617 (1998).

14. T. C. He, T. A. Chan, B. Vogelstein, K. W. Kinzler, PPARdelta is an APC-regulated target of nonsteroidal anti-inflammatory drugs. *Cell* **99**, 335-345 (1999).

15. J. Abramson *et al.*, Accurate structure prediction of biomolecular interactions with AlphaFold 3. *Nature* **630**, 493-500 (2024).

16. L. Schrödinger, in *Schrödinger, LLC*. (2002).

17. L. C. Xue, J. P. Rodrigues, P. L. Kastritis, A. M. Bonvin, A. Vangone, PRODIGY: a web server for predicting the binding affinity of protein-protein complexes. *Bioinformatics* **32**, 3676-3678 (2016).
